# A modular and flexible pipeline for intraoperative electrode reconstruction and localization in patients with brain lesions

**DOI:** 10.3389/fncir.2026.1814667

**Published:** 2026-06-10

**Authors:** Haziq Rabbani, Ipsita Das, Ayan S. Mandal, Thomas Nelson, Peter Hadar, Brian Hsueh, Roberto Ciordia, Brian F. Coughlin, Emory Peng, Daniel R. Cleary, Kelly L. Collins, Ahmed M. T. Raslan, Ziv M. Williams, Bryan D. Choi, Garth Rees Cosgrove, Shadi A. Dayeh, Pamela S. Jones, Harmanpreet K. Tiwana, Gavin P. Dunn, R. Mark Richardson, Wenya Linda Bi, Steven Tobochnik, Sydney S. Cash, Daniel P. Cahill, Angelique C. Paulk

**Affiliations:** 1 College of Medicine, Northeast Ohio Medical University, Rootstown, OH, United States; 2 Department of Neurology, Mass General Brigham, Boston, MA, United States; 3 Center for Neurotechnology and Neurorecovery, Boston, MA, United States; 4 Division of Neuro-Oncology, Department of Neurosurgery, University of California, San Francisco, San Francisco, CA, United States; 5 Department of Neurosurgery, Mass General Brigham, Boston, MA, United States; 6 Department of Neurosurgery, Oregon Health State University, Portland, OR, United States; 7 Department of Neurosurgery, University of California, San Diego, La Jolla, CA, United States; 8 Center for Neuro-Oncology, Department of Medical Oncology, Dana-Farber Cancer Institute, Boston, MA, United States

**Keywords:** electrophysiology, epilepsy, human, imaging, intraoperative, parcellation, tumor

## Abstract

Intraoperative intracranial electrophysiological recordings provide unique access to human cortical dynamics but remain difficult to translate across patients due to inconsistent localization of transient surface electrodes. Unlike chronic implantations, intraoperative electrodes are placed transiently, rarely visible on imaging, and often inconsistently documented. We present an open-source imaging pipeline, ALIGNER (Advanced Localization and Imaging Guidance for Neurosurgical Electrode Recording), designed to reconstruct intraoperative surface electrode array placements and quantitatively map neural activity to individualized anatomical and pathological substrates. By enabling anatomical localization of these electrodes, this framework supports systematic analysis of spatial gradients in neural activity relative to pathological tissue. We developed a multimodal reconstruction framework integrating pre- and postoperative MRI and CT, cortical surface modeling, semi-automated pathology segmentation, intraoperative photographs or videos when available, and physics-based electrode modeling. To improve robustness in cases with distorted anatomy, artificial intelligence tools such as SynthSR were used to enable reliable cortical surface reconstruction prior to FreeSurfer processing. A monocular depth-estimation network was incorporated to constrain electrode placement in conjunction with Blender cloth-physics simulation when photographic images were available, while atlas- and note-guided inference supported reconstruction otherwise. The pipeline was applied to 38 neurosurgical patients across drug-resistant epilepsy resection (*n* = 24), malformation (*n* = 1), brain tumor (*n* = 11), and deep brain stimulation (*n* = 2) cases, achieving some type of reconstruction and electrode localization in all participants. By exporting electrode coordinates for quantitative spatial analyses, including distance-based mapping relative to lesions and resection cavities, ALIGNER enables anatomically grounded and reproducible analysis of intraoperative electrophysiology. This open-source framework provides foundational infrastructure for cancer neuroscience studies of tumor–neuron interactions and establishes a scalable platform for future neurostimulation, implantable neurodevice, and brain–computer interface applications requiring precise anatomical localization.

## 1 Introduction

Mapping neural activity in the human brain requires techniques that combine high spatial resolution with precise anatomical localization. Although non-invasive recording modalities such as functional MRI (fMRI) and magnetoencephalography (MEG) have greatly contributed to the current understanding of brain networks, their spatiotemporal resolution falls short in capturing the fine-scale dynamics that are critical to understanding focal pathologies (Hämäläinen et al., 1993; Logothetis, 2008; Baillet, 2017). Moreover, fMRI offers millimeter spatial detail constrained temporally by hemodynamic responses on the order of seconds, while MEG provides millisecond temporal resolution with limited spatial precision due to volume conduction and depth sensitivity (Hall et al., 2014).

Intracranial electroencephalography (EEG) recordings address these constraints by sampling neural activity with both temporal (millisecond resolution) and spatial (millimeter-scale) precision. As a result, subdural electrocorticography (ECoG) and stereo-EEG (sEEG) represent the current gold standards for analyzing human cortical dynamics (Lachaux et al., 2003; Parvizi and Kastner, 2018). These approaches are indispensable in neurosurgical contexts, where detailed mapping of pathologic tissue margins is necessary to inform resection while preserving eloquent cortex. Beyond their clinical applications, intracranial EEG recordings provide a unique window into understanding network disruptions in neurological disease, such as hyperexcitability in epileptogenic cortex and neuron–glioma synaptic integration at tumor margins (Frauscher et al., 2018; Venkatesh et al., 2019).

Translating intraoperative electrophysiological recordings into anatomical frameworks remains an ongoing challenge. In chronic implantation cases, electrode positions can be reliably reconstructed using post-implant CT and MRI co-registration, and several validated pipelines now exist to standardize this process (Davis et al., 2021; Soper et al., 2023). However, intraoperative surface electrode arrays are placed and removed transiently during surgery, are seldom observable on imaging, and often lack systematic documentation. The development of electrode technologies has advanced rapidly (Paulk et al., 2024), but accurate, reproducible methods for reconstructing intraoperative placements in relation to patient-specific anatomy remain underdeveloped (Oten et al., 2025). This gap limits both clinical interpretation and research into the fine-scale gradients of neural activity surrounding pathology, constraining investigation of spatial gradients in neural activity surrounding pathological tissue and limiting both mechanistic neuroscience insights and the interpretation of intraoperative physiology for surgical decision-making.

In this study, we present ALIGNER (Advanced Localization and Imaging Guidance for Neurosurgical Electrode Recording), a flexible, open-source pipeline for intraoperative surface electrode array localization and anatomical reconstruction. Elements of this pipeline have proven useful for electrode placement in brain-computer interfaces (Hadar et al., 2026), but we wanted to test its flexibility in applying electrode localization in the transient intraoperative space (Kekhia et al., 2011; Rigolo et al., 2020). ALIGNER combines multimodal imaging (MRI and CT), cortical surface modeling, intraoperative photographs, and a physics-based electrode model within Blender (an open-source 3D modeling and physics-simulation software; Community, 2018). Moreover, to enhance the precision of surface electrode array localization, we incorporated MiDaS (Monocular Depth Approximation System), a state-of-the-art depth-estimation neural network originally trained on LiDAR (Light Detection and Ranging) and stereo data (Ranftl et al., 2022); this represents a novel application of the technology in neuroscience contexts to generate depth maps from intraoperative photographs and videos. We used these depth cues to align electrodes to patient-specific cortical anatomy as they existed in the operating room, with cloth-physics simulation providing realistic electrode seating. Notably, the ALIGNER pipeline enables quantitative mapping of electrophysiological signals onto 3D models of pathology, vasculature, and resection cavities.

We further demonstrate the feasibility of this pipeline in a cohort of patients undergoing neurosurgical procedures, including resection for drug-resistant epilepsy (*n* = 25), brain tumors (*n* = 12), malformation (*n* = 1), or undergoing deep brain stimulation (DBS) implantation for movement disorders (*n* = 2). By incorporating segmentation methods, open-source computational tools, and machine learning depth estimation, ALIGNER enables systematic reconstruction of intraoperative anatomy and electrode placement. This work establishes a framework for future investigations into the spatial gradients of neural activity around pathological tissue in intraoperative datasets, with the potential to inform both neuroscience and surgical decision-making.

## 2 Materials and methods

### 2.1 Electrode placement and recordings

Human recordings with intracranially placed electrodes were acquired from 38 participants (mean age: 42 years, range 22–75; 22 female) (Supplementary Table 1) who were either scheduled for surgical resection of cortical tissue as a result of tumor or epilepsy or for deep brain stimulation (DBS) implantation for movement disorders at Massachusetts General Hospital (MGH) and Brigham and Women’s Hospital (BWH). All of the patients (*N* = 36) aside from four were already scheduled for a craniotomy for concurrent clinical intraoperative neurophysiological monitoring or testing for mapping motor, language, and sensory regions and removal of tissue (Berger and Ojemann, 1992; Spena et al., 2010; Simon et al., 2018; You and Qiao, 2021). In two cases, patient participants had two resective surgeries; these participants each appear twice in the supplementary tables, accounting for the 40 entries in Supplementary Tables S1, S2 despite a cohort of 38 unique participants. The remainder (*N* = 2) were scheduled for deep brain stimulation (DBS) implantation for movement disorders (Paulk et al., 2022). All of the included data set outside of four participants have been included in previous publications (Supplementary Tables S1, S2) (Paulk et al., 2021, 2022; Yang et al., 2021; Coughlin et al., 2023; Tan et al., 2023; Lee et al., 2024).

All patients voluntarily participated after providing informed consent according to NIH guidelines as monitored by per-site Institutional Review Boards (IRBs). Massachusetts General Brigham Institutional Review Board (formerly Partners; IRB) provides coverage for Massachusetts General Hospital (MGH) and Brigham and Women’s Hospital (BWH). Participants were informed that participation in the experiment would not alter their clinical treatment in any way, and that they could withdraw at any time without jeopardizing their clinical care.

**Figure 1:**
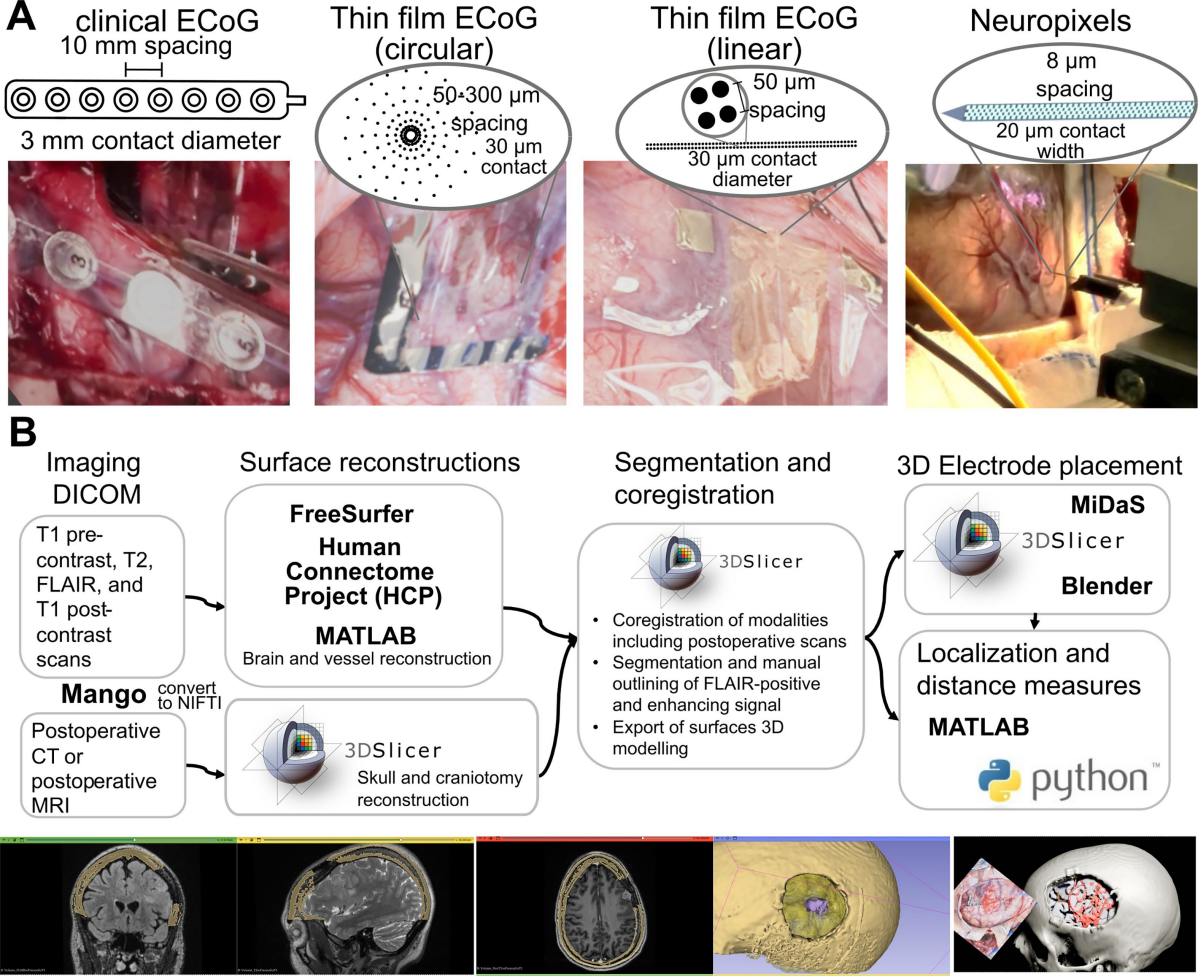
Devices used and the ALIGNER pipeline workflow including software used. **(A)** Electrodes localized to brain regions in this study. **(B)** Overall pipeline.

Importantly, we sampled neural activity using a range of devices which included clinical strips and grids, thin film PEDOT: PSS contacts on a parylene C substrate, and Neuropixels probes (Figure 1A; Paulk et al., 2021, 2022; Yang et al., 2021; Tan et al., 2023). The fabrication of the PEDOT: PSS device is previously published (Uguz et al., 2016; Ganji et al., 2017; Paulk et al., 2021; Yang et al., 2021; Tan et al., 2023). The thin film devices included three different PEDOT: PSS electrode designs. One electrode type was two columns of 64-channel 20 μm diameter contact sites with 50 μm center-to-center pitch between adjacent contacts, which we call the 50 μm pitch 2-column grid (*n* = 19) (Figure 1A; Supplementary Table S2). A second array had the same material make-up but the center-to-center pitch was 800 μm, which we call the 800 μm pitch 2-column grid (*n* = 1) (Figure 1A; Supplementary Table S2). A third array was a 128-channel grid with concentric rings at varying distances between each electrode site, which we call the circular grid ~4 mm in diameter (*N* = 11) (Figure 1A; Supplementary Table S2).

When used, intracranial intraoperative clinical electrocorticography recordings were performed simultaneously using PMT or Ad-tech electrodes (Ad-tech Medical, Racine, WI, United States, or PMT, Chanhassen, MN, United States). In general, at BWH, Ad-tech clinical strip platinum electrodes utilized 10 mm spacing and 2.3 mm contact diameter, while Ad-tech depth platinum electrodes employed 5–8 mm spacing, 2.41 mm contact size, and 1.12 mm diameter. At MGH, PMT Cortac clinical strip platinum electrodes used 10 mm spacing, 3 mm contact diameter. The neurosurgeon placed clinical electrodes in the regions of clinical interest.

Neural recordings included the Intan system (Intan Corporation, Los Angeles, CA, 30 kHz sampling rate, using the RHD2000 or OpenEphys series software), the IMEC Neuropixels Chassis and board (Paulk et al., 2022) the Neural Signal Processor (Blackrock Microsystems, United States). A clinical Quantum system recorded neural activity at 512, 1,024, 2,048, or 4,096 Hz for the clinical strips (Paulk et al., 2021, 2022; Yang et al., 2021; Tan et al., 2023). Spectral curves were calculated using both the Morelet wavelet transform at a 1-Hz spectral resolution from 0 to 200 Hz using the Fieldtrip tools (Oostenveld et al., 2011).^1^

### 2.2 Imaging acquisition and preprocessing

**Figure 2:**
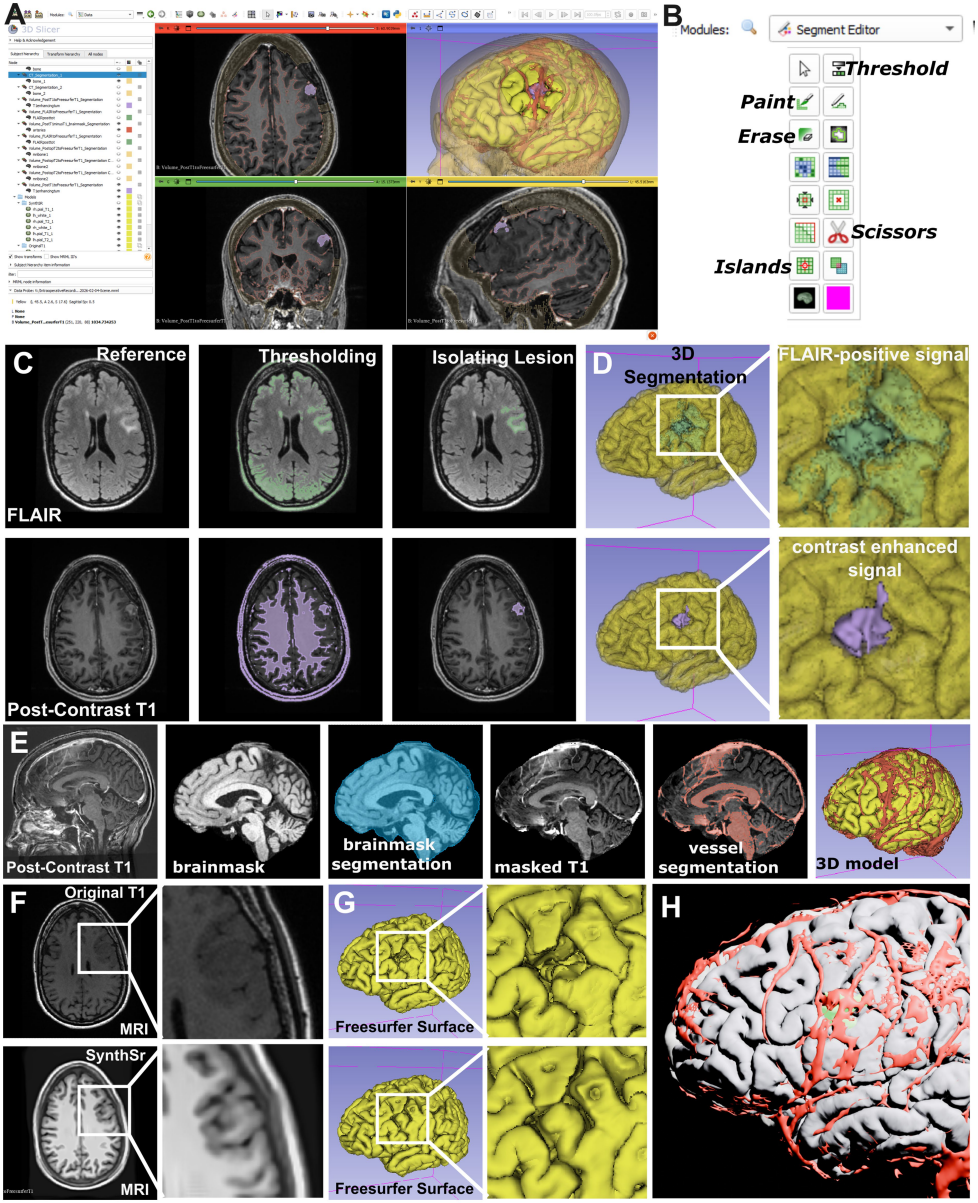
Overview of the structural brain, skull, and neuropathology reconstruction pipeline for IP35. **(A)** 3D Slicer view of post-contrast T1 with outlined tumor and vasculature along with the craniotomy and FreeSurfer 3D model. **(B)** Useful interfaces used with segmentation. **(C)** Left to right steps of pathology segmentation: Representative axial MRI slice used as reference for edema (green, top; FLAIR) or tumor (purple, bottom; Post-contrast T1 used) segmentation in 3D Slicer. Initial semi-automated edema segmentation generated by thresholding hyperintense regions. Axial slice following manual, slice-by-slice refinement of edema or tumor segmentation boundaries. **(D)** Final three-dimensional edema (top, green) or tumor (bottom, purple) model. **(E)** Left to right steps of vascular segmentation: Mid-sagittal MRI slice of the subtraction volume generated by subtracting the pre-contrast T1-weighted scan from the post-contrast T1-weighted scan, highlighting contrast-enhancing vessels. FreeSurfer-derived brain mask (brainmask.mgz) from the reference post-contrast T1-weighted image imported into the 3D Slicer scene. Initial whole-volume segmentation created using the Threshold function to capture all non-zero voxels and exported as a binary label map. Masked subtraction volume generated using the Mask Scalar Volume module, with the subtraction volume as the input and the binary label map as the mask. Final vessel segmentation obtained through manual threshold adjustment to isolate hyperintense vascular structures. Three-dimensional reconstruction of vasculature, used as fiducial landmarks for electrode localization. **(F)** Axial slice with noted tumor-related discoloration of the scan (top). Axial slice following preprocessing with SynthSR (bottom). Note the difference at the site of the tumor. **(G)** Final cortical surface model generated by FreeSurfer before (top) and after SynthSR (bottom) preprocessing, showing improved cortical continuity. **(H)** 3D reconstruction of brain and vessels with the tumor in Blender.

To map electrode locations to brain regions and pathologies, we used a multi-platform pipeline (Figure 1B; code available at https://github.com/Center-For-Neurotechnology/BrainInterface3D). Following the surgery, the preoperative T1-weighted MRI was reconstructed using FreeSurfer scripts (Desikan et al., 2006; Reuter et al., 2010, 2012; Dale et al., 1999; Fischl and Dale, 2000; Fischl et al., 2001; Figures 1, 2)^2^. In a subset of cases and for further surface validation, we also reconstructed surfaces using the Human Connectome Project pipeline (Glasser et al., 2013, 2016; Elam et al., 2021). ^3^ The steps involved obtaining deidentified magnetic resonance imaging (MRI) and computed tomography (CT) scans in Digital Imaging and Communications in Medicine (DICOM) format for each patient. These were converted to NIfTI formats for the preoperative, intraoperative, or postoperative MRI sequences, including T1-weighted, T2-weighted, fluid-attenuated inversion recovery (FLAIR), and post-contrast T1-weighted images (after intravenous gadolinium administration). Where possible, postoperative CT scans were converted from DICOM to NIfTI, acquired to visualize skull anatomy and the craniotomy boundaries. The extraction of imaging scans, conversion to NIfTI format, and generation of three-dimensional cortical models using FreeSurfer 7.4 followed previously published procedures (Figures 2A,D,G). All DICOM imaging scans were converted to NIfTI format using the Mango v4.1^4^ command-line utility (mango-convert2nii) or dcm2niix.^5^ Example commands used are available at https://github.com/Center-For-Neurotechnology/BrainInterface3D.

T1-weighted and T2-weighted scans were input into FreeSurfer’s recon-all pipeline to reconstruct cortical and subcortical surfaces, resulting in automated segmentation of white and gray matter and reconstruction of pial surfaces (Figures 2A,D,G). The resulting FreeSurfer models were used as the three-dimensional cortical surfaces onto which electrode representations were mapped (Desikan et al., 2006; Reuter et al., 2010, 2012).

FreeSurfer may generate cavities during the cortical reconstruction process in the presence of large lesions or distorted brain anatomy due to pathology (Figure 2D). In such cases, SynthSR was applied prior to FreeSurfer processing.^6^ SynthSR converts clinical MRI or CT scans of varying orientation, resolution, and contrast into an isotropic 1-mm MP-RAGE-like image suitable for anatomical modeling (Iglesias et al., 2021, 2023). By replacing lesion regions with anatomically consistent tissue contrast, SynthSR enabled FreeSurfer processing to reconstruct continuous cortical surfaces and parcellate the surfaces into brain regions with the DKT atlas. In some cases, the pre-contrast T1 images were not high resolution, while the post-contrast T1 images were of higher resolution. In order to capture the reconstructed surface at higher resolution, SynthSR was applied to the post-contrast T1 to then run through surface reconstruction in FreeSurfer. In a few cases, we were able to use SynthSeg, an ML tool for segmenting brain regions (Billot et al., 2021, 2022, 2023b),^7^ which allowed us to map parcellated brain regions to problematic T1 scans. This was done as the original recon-all FreeSurfer parcellations had some errors due to previous resections. In addition, SynthSeg allowed for near-instant surface reconstruction and parcellation, bypassing the slower FreeSurfer workflow (Billot et al., 2021, 2023a, 2023b).

On the relative roles of the Human Connectome Project (HCP) and FreeSurfer 7.4 pipelines, we found that HCP reconstructions were generally superior at recreating the cortical surface when scans were of high quality and free of large dural or surface defects, with clearer delineation of gyri and sulci visible when the reconstructed surface was overlaid on the MRI in 3DSlicer (Kikinis and Pieper, 2011; Fedorov et al., 2012). However, HCP frequently either failed to complete processing or produced visibly poor reconstructions when T1 or T2 image quality was reduced or when the lesion or surgical defect was sufficiently large to disrupt the pipeline. In these cases, FreeSurfer 7.4 was more tolerant and sometimes generated visibly accurate surfaces where HCP did not, although it more often required manual correction. The choice between pipelines was therefore made on a per-case basis according to imaging quality and the extent of pathology. SynthSR and SynthSeg was applied in the smaller subset of cases described above, specifically when recon-all parcellations contained errors attributable to prior resections, or when rapid surface reconstruction and parcellation was required in place of the longer FreeSurfer workflow.

### 2.3 Co-registration and anatomical model construction

FreeSurfer surface outputs (lh.pial. T1, lh.pial. T2, lh.white, rh.pial. T1, rh.pial. T2, and rh.white) were imported into 3D Slicer v5.8.1 (as well as 5.6.2 and other 3D Slicer versions) as *FreeSurfer models* which was also enabled with the SlicerFreeSurfer add-on (Figures 2A,D,G).^8^ Selected T1-weighted, T2-weighted, FLAIR, and post-contrast T1-weighted NIfTI scans were imported as *Volumes* (Figures 2A, C). All scans were co-registered to the T1-weighted image (either the original acquisition or the SynthSR-generated output) used as input to FreeSurfer’s recon-all pipeline. Co-registration was performed using the General Registration (ANTs) module,^9^ with the T1-weighted image specified as the *Fixed Image* and each additional scan specified as the *Moving Image*. Registration stages (presets) were defined as Rigid + Affine. This module produced a transform and a transformed volume for each co-registered scan so that all scans were in the same co-registered space. The transformed volumes were used for all downstream segmentations. Following co-registration, all surface reconstructions were visually inspected in 3D Slicer by comparing the registered surfaces against the underlying imaging volumes before proceeding to subsequent reconstruction steps, providing a case-by-case quality check on surface accuracy.

### 2.4 Neuropathology segmentation

Lesion, resection cavity, and edema segmentations were performed in 3D Slicer using a semi-automated workflow (Figures 2A–C,E). The *Thresholding* function was applied to co-registered images to identify regions of interest. Threshold ranges were manually adjusted to capture pathological tissue and applied to generate the initial segmentation. For non-post-contrast T1 or FLAIR iso- or hyperintense lesions such as mesial temporal sclerosis, this step was skipped. Manual slice-by-slice editing was performed to refine segmentation boundaries using the *Eraser* and *Scissors* functions. The *Islands* function was additionally used to remove small, isolated voxel clusters of noise that were not connected to lesions of interest and not anatomically consistent with pathology. All segmentations were validated and further refined by neurologists and neurosurgeons (RC, PH, BH, TN, ST) to ensure anatomical and clinical accuracy.

For the labels of “tumor” or “lesion in non-tumor epilepsy,” we relied on the input from neurologists and neurosurgeons to identify the specific label. This required knowledge of the tumor type or lesion type as well. For instance, for IDH-wild type glioblastomas, most of the cases had tumor tissue expressing as enhancing post-contrast T1 voxels in the MRI scans. IDH-mutant cases, on the other hand, generally had FLAIR-hyperintense tissue which was segmented and outlined manually. We also used the FLAIR or T2 scans to identify edema boundaries and post-contrast T1 sequences to identify necrotic regions in the tumor center. Previous resections were segmented based on comparing all scans, particularly regions which were fluid-filled. Final pathology segmentations were exported as models for subsequent modeling steps.

### 2.5 Craniotomy and vasculature reconstruction

**Figure 3:**
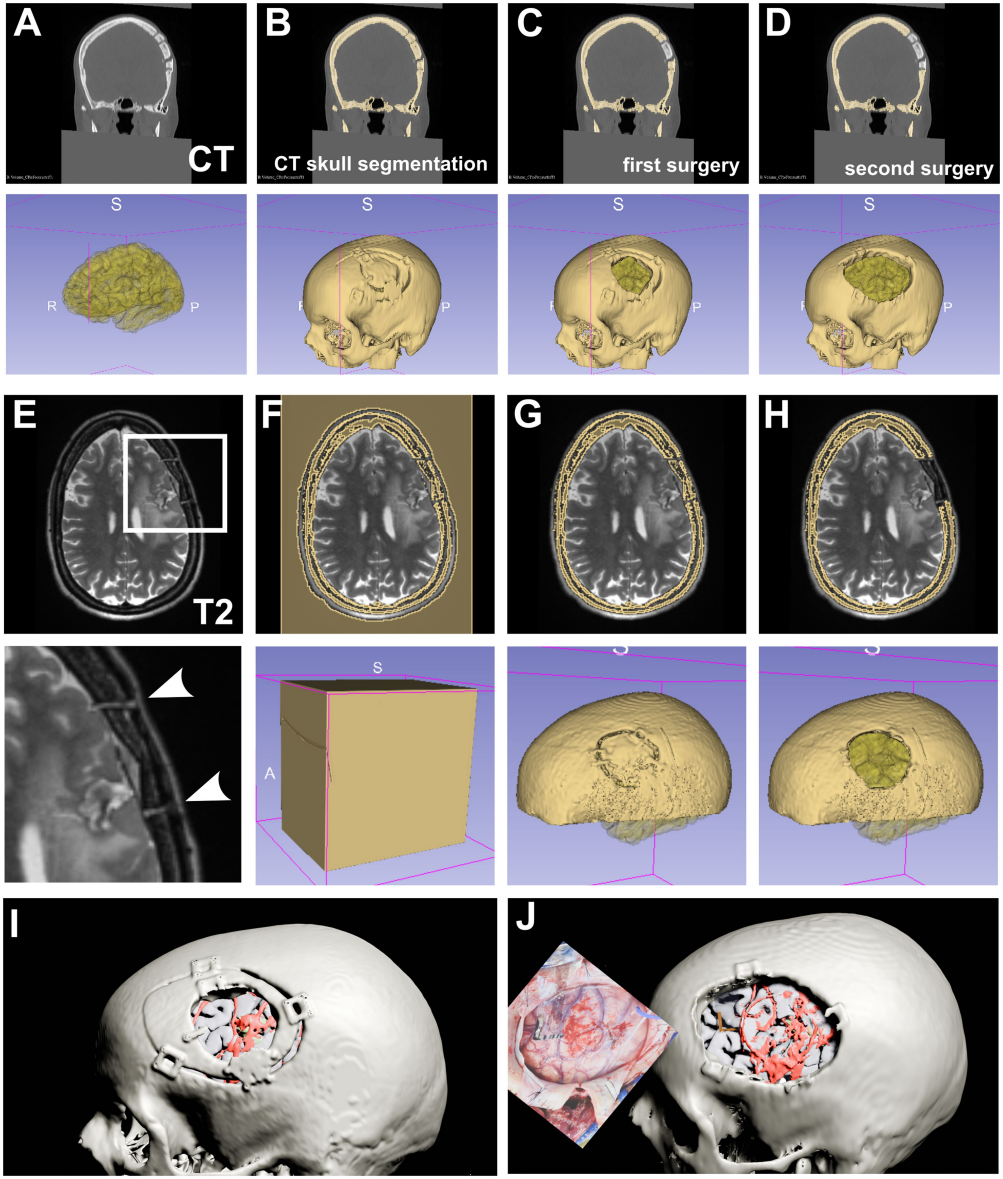
Craniotomy reconstruction from postoperative CT for IP35. **(A–C)** Initial skull segmentation using the threshold function to isolate hyperdense bone with manual threshold adjustment (top), and resulting 3D skull reconstruction (bottom). Reconstruction of the first craniotomy. Craniotomy boundaries were identified as subtle hypodense lines on CT, and the skull flap was manually removed using slice-by-slice editing to generate the craniotomy model. **(D)** Reconstruction of the second craniotomy using the thresholding and manual editing approach described in **(A–C)**. **(E)** Axial postoperative T2 MRI slice (top) with zoomed-in view and arrowheads pointing to postoperative evidence of the craniotomy. **(F–H)** Initial skull segmentation using inverse thresholding to isolate hypointense skull regions with manual threshold adjustment, and resulting 3D reconstruction. The skull segmentation was manually refined slice-by-slice in 3D Slicer (Eraser and Scissors tools) to remove artifacts and improve skull boundaries, producing a cleaned 3D skull surface, with artifact removed using slice-by-slice editing to generate the final craniotomy model. **(I,J)** Blender files of the resulting craniotomy with vasculature and brain reconstruction for the first and second surgery.

Craniotomy reconstruction was performed in 3D Slicer using a combination of threshold and manual editing tools (Figure 3). For cases with available postoperative CT scans, the *Threshold* function was applied to isolate the skull, with the threshold range manually adjusted to highlight hyperdense regions of bone. For CT scan reconstructions, the skull flap and screws from the craniotomy were also reconstructed via thresholding. The intraoperative skull incision boundaries were identified as subtle hypodense lines on the CT slices; the skull flap was then manually removed from the 3D skull model using slice-by-slice editing with the *Eraser* and *Scissors* functions.

For cases without available postoperative CT imaging, inverse thresholding was applied to T1-weighted or T2 postoperative or intraoperative MRI to isolate hypointense skull regions. The threshold range was manually adjusted to generate an initial skull segmentation. Manual slice-by-slice editing was then performed using the *Eraser* and *Scissors* functions to remove imaging artifacts and refine skull boundaries (Figures 3B,C,F,G). The craniotomy site was identified as slightly hyperintense lines in the MRI slices, marking the incision boundaries (Figure 3E). This region is made apparent due to contents such as edema, blood products, or tissue partially filling the surgical defect, making the craniotomy boundary visible. The flap was manually removed from the 3D skull model using slice-by-slice editing with the *Eraser* and *Scissors* functions to reconstruct the craniotomy.

The brain vasculature was reconstructed to provide fiducial landmarks for electrode localization (Figures 2E,H, 3I,J). The Subtract Scalar Volumes module was used to subtract the non-contrast T1 from the post-contrast T1, producing an output volume that highlighted enhancing vessels. The brainmask.mgz output from FreeSurfer was added to the 3D Slicer scene as a reference volume, and the *Threshold* function was applied to segment the entire scan area, capturing all non-zero voxels. This initial segmentation was exported as a binary label map and subsequently used as the mask in the Mask Scalar Volume module, with the T1 subtraction volume serving as the *Input Volume* and the label map as the *Mask Volume*, producing a masked output volume. This masked volume was further segmented using the *Threshold* function, with threshold ranges manually adjusted to isolate vessels, which appeared bright in the scan (Figure 2E).

The resulting skull and vasculature segmentations were exported as models (Figures 3I,J). All models generated from the segmentations of lesions, skull, and vasculature were saved in PLY format, a format which allows transfer of points and surfaces between platforms including color coding of different brain regions. Exporting the FreeSurfer brain surface with color coding was enabled through the import of surfaces into MATLAB, the addition of color coding using the surfacemesh function, and the export of the mesh as a PLY file with writeSurfaceMesh (code available at https://github.com/Center-For-Neurotechnology/BrainInterface3D). Since it is critical to have all these models and surfaces registered to the T1 that all imaging is registered to, the color-coded FreeSurfer surfaces were then imported into 3Dslicer and then moved (via a transform operation) to match the location of the coregistered T1.

### 2.6 Electrode localization and reconstruction

**Figure 4:**
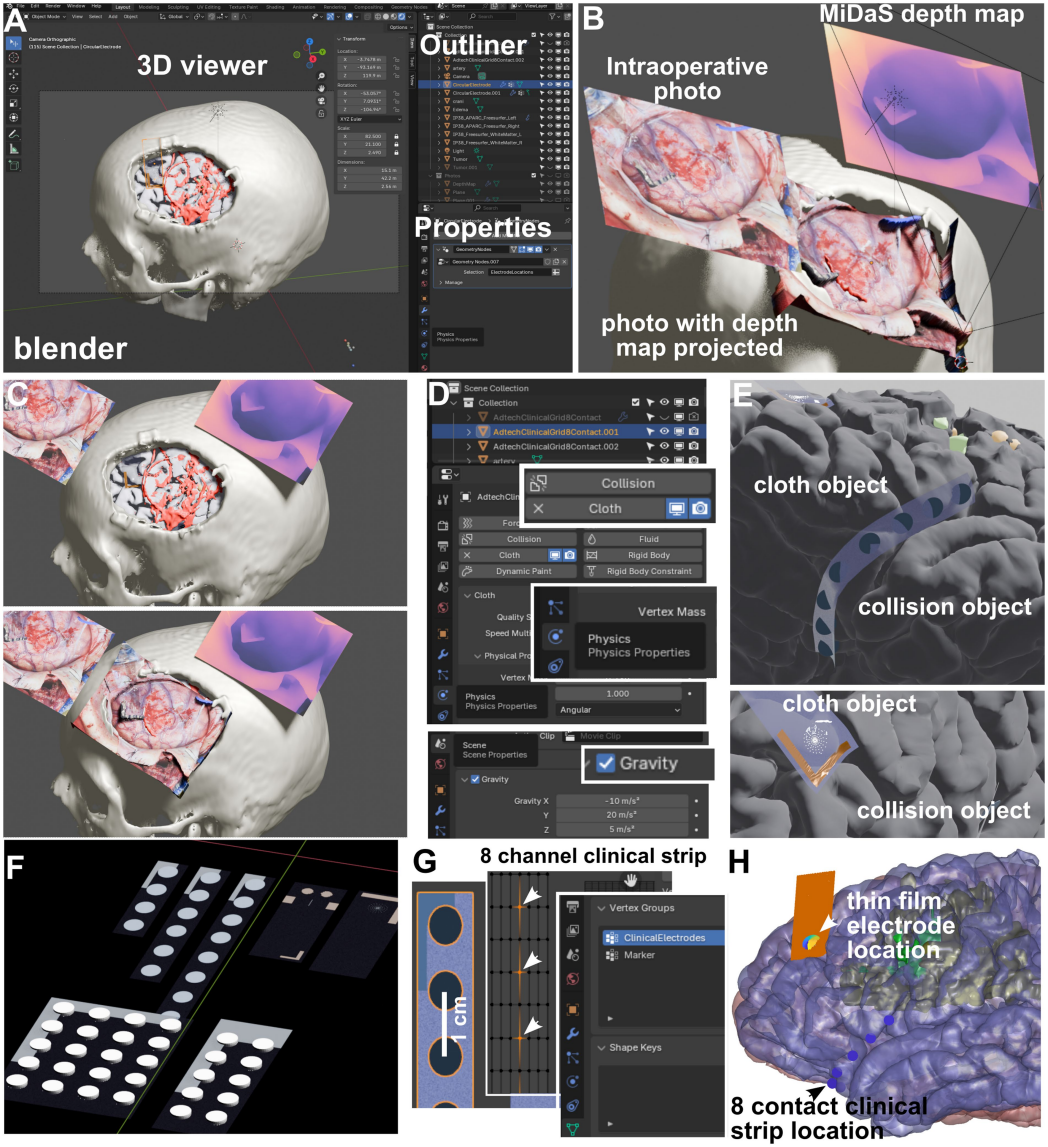
Electrode placement and map projection from photographs for IP35. **(A)** Screenshot of the Blender file with imported PLY surfaces for the reconstruction with a view of the imported vessels and craniotomy. **(B,C)** Craniotomy view with planes on which the surgical photographs are projected with an image of the MiDaS depth map (Ranftl et al., 2021, 2022) which then was projected onto a subdivided plane with the corresponding photograph and enabled different depths showing the extent of the curvature of the craniotomy which allowed for a side by side comparison of the 3D model and the photograph. **(D,E)** In **(D)**, the gravity values are only for the sake of illustration for a particular case, not what is used for all cases. Panels in Blender which allowed for the use of cloth physics to curve the 3D model electrodes around the brain regions, treating the brain as a collision object and the electrode as a cloth object, with “gravity” redirected to allow for the curvature. **(F)** 3D modeled electrodes. **(G)** 3D model 8-channel strip created to scale relative to the 3D model, which is made up of faces and vertices. Specific vertices are selected and labeled as clinical electrodes. After the cloth step **(D,E)**, the vertex points in RAS space are exported with Python code into .csv files which can then be imported into other programs such as MATLAB to measure distance to pathology or map physiological signals. **(H)** Indicated are the locations of the 128 channel thin film electrode (white arrow, yellow, blue and green dots) and the 8-contact clinical strip (black arrow, blue dots).

The critical step was to then import the models generated in 3D Slicer segmentations into a space which would allow us to coregister electrode devices relative to captured photographs or documentation.^10^ Following segmentation and 3D reconstruction in 3D Slicer, the PLY models of the brain (lh.pial. T1, lh.pial. T2, lh.white, rh.pial. T1, rh.pial. T2, and rh.white), lesion, vasculature, and skull were imported into Blender for electrode localization (Figure 4A; see text footnote 10).

For cases with intraoperative videos and photographs documenting surface electrode array placement (*n* = 33), surface electrode arrays were positioned manually to match surgical views. The video recordings were acquired by a camera set in the operating room light (Black Diamond Video). To enhance the precision of this process, we leveraged MiDaS,^11^ a depth-estimation ML library originally trained on LiDAR and stereo data (Ranftl et al., 2021, 2022). We used the pretrained DPT-Large model on our intraoperative photos and videos, which generated depth maps that, when overlaid onto the brain models, provide surface shape constraints used to guide electrode drape; absolute scale and spatial registration are resolved by alignment to the patient-specific craniotomy model derived from postoperative CT. These depth estimates could then be projected onto a subdivided plane in the Blender file and adjusted for depth to match the 3D reconstructed craniotomy and exposed brain (Figures 4B,C; see text footnote 10). This helped to visualize the location of objects relative to each other in the intraoperative space.

**Figure 5:**
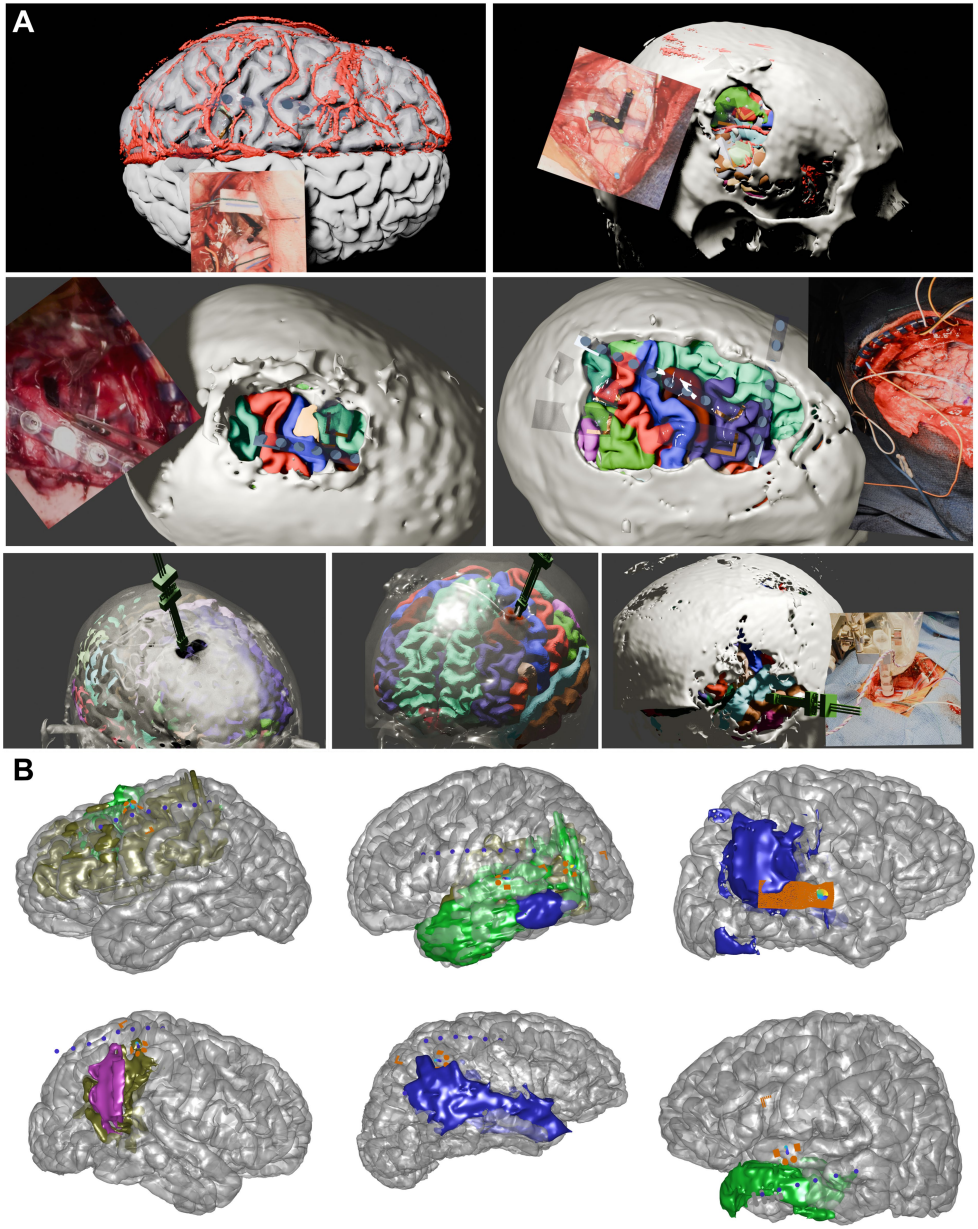
Example segmentations and 3D localization of electrodes relative to craniotomies, lesions, and parcellations. **(A)** Blender 3D reconstructions, including brain regions color-coded based on the DKT atlas (Desikan et al., 2006). **(B)** Tumor (green), previous resection (blue), lesion in lesional epilepsy (pink), edema (tan), and brain surface (grey) localization relative to various devices placed on the brain.

For cases lacking video or photos (*n* = 7), clinical strip location was deduced from intraoperative monitoring (IOM) notes and FreeSurfer’s APARC (automatic parcellation using the DKT atlas) atlas labels, aligning contacts to cortical regions as specified by the clinicians (Figure 5A). IOM documentation was reviewed for written descriptions and schematic diagrams or photographs indicating strip orientation relative to anatomical landmarks. Although diagrams and photographs were not consistently included in the documentation, each case contained written descriptions and/or stimulation findings sufficient to localize electrodes to a specific cortical region. Task-evoked IOM responses such as identification of the location of the central sulcus from motor mapping were also cross-referenced with APARC-defined cortical regions to constrain electrode placement to anatomically and functionally plausible locations.

Finally, electrode localization involved referencing photos or videos taken intraoperatively which included the craniotomy and the electrode and then placing a 3D model of the electrode onto the patient’s reconstructed brain in Blender. Building these devices involved adding per-vertex labels to the model which then could be exported into RAS coordinates (with the patient right, anterior, superior coordinate system) as “electrode” points relative to the coregistered MRI (Figure 4G; see text footnote 10). This approach allowed us to generate multiple models of electrodes easily for 3D-modeled placement using the Physics engine in Blender.

A cloth-physics and gravity model (“dropped” electrode array) was used to simulate intraoperative electrode seating (Figures 4D,E). Scene parameters were configured to mimic intraoperative conditions, including setting the direction of gravity along the appropriate axis in the Scene Properties feature so that the electrodes would fall in the correct orientation. The pial surfaces of the brain were designated as collision objects within the Physics feature (Figures 4D,E). Surface electrode arrays were modeled using Blender’s Cloth Simulation tool, allowing adjustment of material properties such as stiffness, damping, and other relevant properties to closely approximate the physical behavior of the electrodes. Cloth material properties were calibrated per case to match the physical behavior of the electrode substrate; structural stiffness, shear stiffness, bending stiffness, spring damping, and vertex mass were each adjusted iteratively until the simulated strip conformed to the cortical surface without unrealistic folding or lifting. The gravity vector direction was set empirically per case rather than using a standard anatomical axis, because the strip orientation during recording is determined by the surgical approach rather than absolute anatomical direction. Components were adjusted until the simulated strip fell onto the cortical surface with plausible contact behavior (e.g., *X* = −10, *Y* = 20, *Z* = 5 m/s^2^ for the reference case IP35). The simulation was paused and hardened when reconstructed contact positions aligned with the photographic, schematic, or note-based record. These adjustments minimized unrealistic stretching and ensured that electrode contacts conformed naturally to the cortical surface. Once the simulation parameters were adjusted, the surface electrode arrays were manually positioned in 3D space and animations were played to “drop” them onto the brain surface, simulating intraoperative placement (Figure 4E). Finally, using a Python script (see text footnote 10), the RAS coordinates of the electrode contacts were exported from Blender for integration with pathological localization data and eventual mapping to electrophysiology in MATLAB (Figure 4E; Supplemental Figure 1). A channel map calibrated to these vertices was used in MATLAB to number the points on a per-channel basis.

For rigid electrodes such as the Neuropixels probe, we acquired a 3D model from the manufacturer, IMEC, scaled it to actual size, added holders as we would use in the operating room, and then imported the 3D model into the Blender file, rotating and placing it in the same or similar location as observed in the photographs or within the burr hole for the eventual DBS electrode (Paulk et al., 2022; Coughlin et al., 2023). For DBS cases specifically, the cloth simulation pipeline described above is not applied, as DBS leads have a fundamentally different geometry (rigid, cylindrical, with defined contact spacing) and are inserted along a stereotactically planned trajectory rather than draped across a cortical surface. Instead, the implanted lead trajectory was derived from the postoperative CT and registered to the preoperative MRI surface model. Contact positions were then calculated from the known lead geometry (contact diameter, spacing, and total array length as specified by the manufacturer) applied along the registered trajectory. A to-scale 3D model of the Neuropixels probe was then placed along that trajectory as a rigid body, with placement performed in both 3D Slicer and Blender. This allowed electrode coordinates to be exported in RAS and MNI space using the same downstream pipeline as for surface arrays.

Once the RAS coordinates were captured relative to the reference T1, MNI space coordinates were calculated using Fieldtrip tools (Oostenveld et al., 2011),^12^ with code made available (see text footnote 10). Contacts appearing to float above or below the brain surface in Figure 7A reflect either electrode contacts that were not in contact with the brain intraoperatively, or residual surface-volume registration discrepancies in perilesional regions; no electrophysiological analyses were performed using these coordinates.

### 2.7 Distance and localization measures

The RAS coordinates of the contacts were imported along with the PLY surfaces of the brain and pathologies (e.g., tumor, lesion, etc.). For the Euclidean distances, surfaces were then treated as point clouds to identify distances between electrode contacts and the pathologies in 3D space. For geodesic distances which account for the distance across the curvature of the brain, we used pymeshlab^13^ following re-meshing the surfaces using MATLAB surfaceMesh and writeSurfaceMesh (see text footnote 10). To identify what brain regions were sampled with the placement of the electrode, we identified the closest brain region labeled vertex (labeled using the DKT atlas in FreeSurfer) to the individual electrode channel contact and labeled that contact as in that brain region.

### 2.8 Reconstruction confidence index

To characterize reconstruction quality across the cohort in the absence of a gold-standard ground truth, we developed a two-domain Reconstruction Confidence Index (RCI). Domain A is scored for all cases and captures surface reconstruction quality.

Criteria included successful completion of FreeSurfer recon-all without topological errors, and whether preprocessing with SynthSR or SynthSeg was required, which was noted as a quality modifier. Within 3D Slicer, evaluation included successful co-registration of all scans to the reference T1 image, generation of skull and craniotomy models from postoperative MRI or CT, reconstruction of vasculature, and completion of lesion segmentation. Within Blender, reconstruction quality was further assessed by electrode placement with respect to identifiable surface features, and the successful export of electrode RAS and MNI coordinates.

Domain B or Domain C is then scored depending on reconstruction pathway. Domain B applies to photo-guided cases (*n* = 33) and assesses intraoperative imaging quality: items include whether intraoperative photographs were present, whether video was available, whether multiple viewing angles were captured, photographic image quality (focus, exposure, field of view), and whether anatomical landmarks (gyral boundaries, sulci, vasculature) were identifiable in both the photograph and the coregistered 3D model, and a photo-model concordance subsection assessing whether vasculature patterns were anatomically consistent with intraoperative images, whether craniotomy edges were clear and consistent with intraoperative images, and whether a MiDaS depth map was available and applied. Domain C applies to note-guided cases (*n* = 7) and assesses documentation quality: items include whether IOM notes were present, whether a hand-drawn schematic was included, whether task-evoked or stimulation-based placement information was available, and whether electrode placement was anchored to an identifiable anatomical surface feature. Domain B and C scores are reported separately rather than as a pooled composite, because photo-guided and note-guided cases are not equivalent in achievable spatial precision: photo-guided cases can achieve contact-level resolution when imaging quality is high, whereas note-guided cases are bounded by schematic fidelity and yield parcellation-level or regional anatomical assignments. RCI scores per case are reported in Supplementary Table S2 and Supplementary data sheet 1.

## 3 Results

### 3.1 Cohort overview

This study was approved by the Massachusetts General Brigham Institutional Review Board covering both Massachusetts General Hospital and Brigham and Women’s Hospital. We enrolled 38 patients undergoing neurosurgical procedures (*n* = 24 drug resistant epilepsy; *n* = 1 malformation; *n* = 11 brain tumors; *n* = 2 Parkinson’s Disease) at Massachusetts General Hospital and Brigham and Women’s Hospital (Supplementary Table S1). With the exception of four participants, all data included has been previously published (Paulk et al., 2021, 2022; Yang et al., 2021; Coughlin et al., 2023; Tan et al., 2023; Lee et al., 2024). Clinical electrode strip recordings were acquired intraoperatively, with strip placement and clinical recording decided by the clinical team. Other electrodes include thin film devices enabled high resolution sampling, curving around the surface of the brain or Neuropixels probes affording 100 s of channels of recording along a thin 70 × 100 μm silicon probe (Figure 1; Uguz et al., 2016; Ganji et al., 2017; Jun et al., 2017; Dutta et al., 2019; Paulk et al., 2021; Yang et al., 2021; Chung et al., 2022; Coughlin et al., 2023; Tan et al., 2023). The ALIGNER pipeline was successfully applied to all patient participants. No cases were excluded from reconstruction. Cases that would otherwise have failed due to prior resection cavities, surgical distortion, or low-resolution imaging were rescued through pathway-specific strategies: SynthSR was applied to enable FreeSurfer surface reconstruction where recon-all failed, and for cases lacking intraoperative photographs, reconstruction proceeded via clinical IOM documentation, hand-drawn schematics, and atlas-guided parcellation. All but one patient had complete MRI datasets (voxel spacing between 0.4 and 4.95 mm, with 3 cases at 1.5 T magnetic strength and 36 at 3 T), and a subset had post-operative CT scans (Supplementary Table S3). Intraoperative photos were available in 33 cases, enabling direct verification of electrode positioning (Supplementary Table S2).

Reconstruction Confidence Index scores were computed for all cases and are reported per case in Supplementary data sheet 1 and Supplementary Table S2, stratified by reconstruction pathway (photo-guided, Domain B; note-guided, Domain C). This structured approach leverages open-source tools (Figure 1) to produce high-fidelity anatomical and electrode reconstructions, enabling quantitative mapping of electrode placement for future analysis of electrophysiological signals related to patient-specific anatomy and pathology. We included a variety of devices and types of cases to highlight the fact that the ALIGNER pipeline is flexible and can be adapted for any resected brain lesion with recording electrodes (Figure 1). To detail the pipeline, we outline the steps using a single example, IP35, which included two different resective tumor surgeries (Figures 2–4).

### 3.2 Reconstruction outputs and timelines for different users

**Figure 6:**
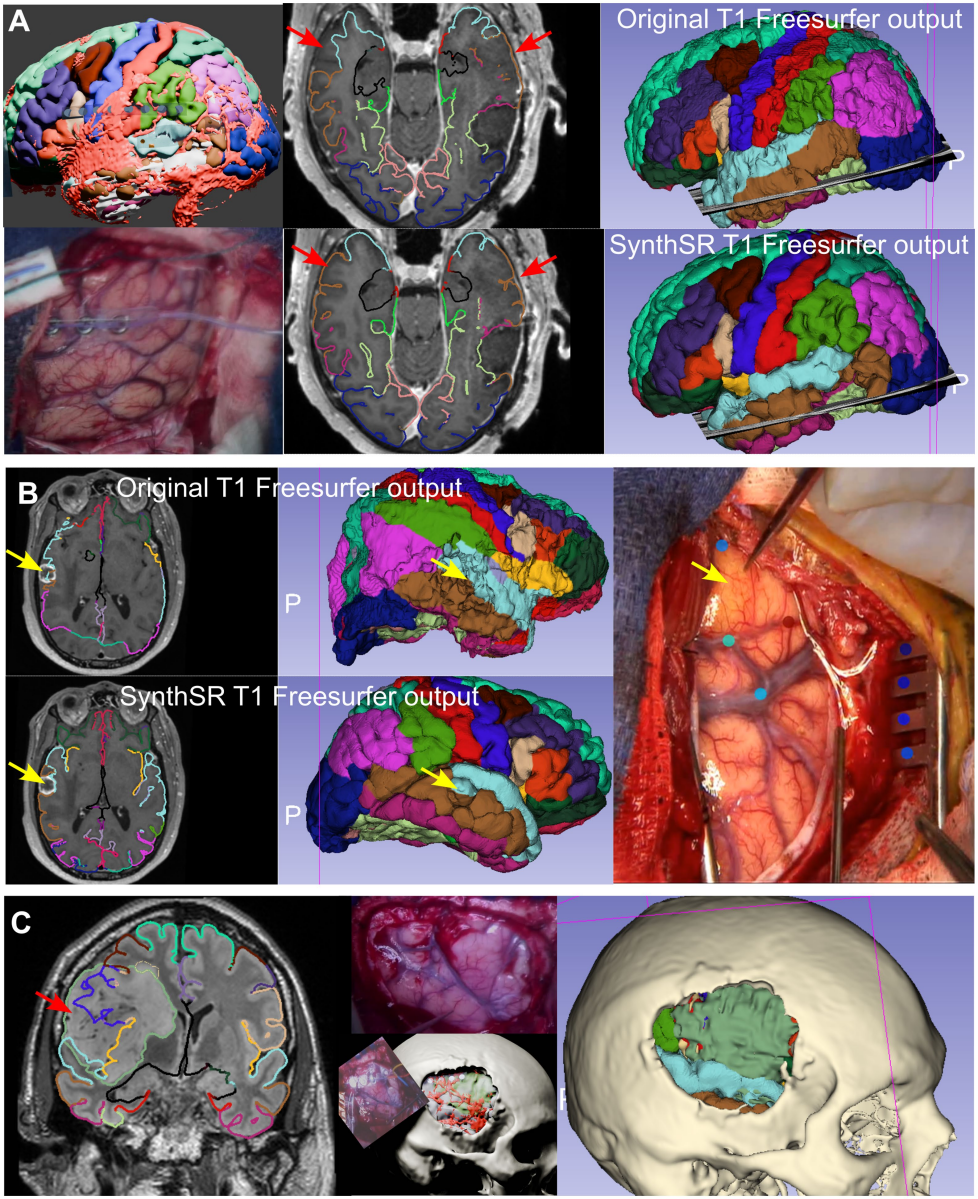
Surface reconstructions between different pipelines. **(A)** IP07 reconstruction from the original pre-contrast T1 scan (top right) versus the higher resolution SynthSR output from the post-contrast T1 (bottom right). Note the red arrows showing the outlined brain surface missing cortex. Right: 3D reconstruction (top) and an image of the continuous, and intact, pre-resection cortical surface. **(B)** IP11 reconstruction with the same original T1 FreeSurfer reconstruction showing multiple defects which were improved with the SynthSR output from the post-contrast T1. The tumor defect (yellow arrow) remained, though there is an intact surface in the original photograph (right). **(C)** IP19 reconstruction where SynthSR FreeSurfer output could not reconstruct the intact surface (middle photograph). The tumor segmentation, however, could be used to “fill in” the cavity.

High-quality cortical surface and lesion models were generated for every patient. In some cases, however, the original T1 scan was lower in resolution or had some motion artifact, resulting in deformed surface reconstructions that are not present in higher resolution scans (Figures 6A–C). The use of SynthSR followed by FreeSurfer reconstructions to better match the imaging as well as the surface photographs of the brain allowed us to reconstruct the brain surface without as many defects. Further, tumor segmentation in cases where the FreeSurfer output produced gaps allowed us to reproduce what is observed in the photographs of the brain: an intact surface (Figures 6A,B). Epilepsy cases showed diverse pathologies like hippocampal sclerosis, neurofibromatosis, and malformations of cortical development, while tumor cases demonstrated sharply demarcated FLAIR/T2 hyperintensity and post-contrast enhancement (Figure 2). Segmentation accuracy was confirmed through manual review by neurologists and neurosurgeons. In a second step, we used the post-contrast T1 to derive the vasculature (Figure 2). The vasculature offered consistent landmarks to orient the photographs (Figure 2).

A third critical step was capturing and reconstructing the craniotomy, particularly as the craniotomy margins constrained electrode placement to a relatively confined surface area (Figure 3). We used the postoperative CT or MRI to then reconstruct the skull with the craniotomy using 3D Slicer segmentation tools (Figure 3). This step then allowed for orienting landmarks in the craniotomy that could be compared with photographs and reconstructed electrode locations.

To characterize the time investment required for the 3D Slicer-based reconstruction steps, three users with no prior experience with 3D Slicer or neuroimaging software were each given a single 30-min demonstration and then asked to independently complete the reconstruction pipeline for a representative case. The demonstrated workflow included image co-registration, lesion, craniotomy, and vasculature segmentation, and export of anatomical models from 3D Slicer in preparation for Blender. The workflow reflected only the manual reconstruction and modeling stages performed in 3D Slicer and did not include the time required for FreeSurfer surface generation, other automated preprocessing steps, or electrode localization in Blender. The three users, all naive to the software and performing the workflow for the first time, completed the reconstruction in 55, 60, and 67 min, respectively. By comparison, an experienced user completed the same reconstruction in under 20 min. Reconstruction time also varied with scan quality, the extent and visibility of the underlying lesion or deformity, and the availability of postoperative imaging suitable for craniotomy and vasculature reconstruction.

### 3.3 Electrode localization and the reconstruction confidence index

Surface electrode arrays were reconstructed in all patients. In cases with intraoperative photographs or video or both modalities, depth maps derived from MiDaS improved alignment between visual documentation and cortical models, producing realistic seating of the electrode arrays (Figures 5A,B). In cases without photos, electrode positions were estimated using intraoperative notes and atlas-based mapping. Of the three experienced users, electrode placement could take 5–15 min depending on the photograph quality.

Reconstruction Confidence Index scores stratified the cohort into interpretable confidence tiers (Supplementary data sheet 1; Supplementary Table S2). Note-guided cases (Domain C, *n* = 7, no photographs included) yielded parcellation- or lobe-level anatomical assignments and were classified as low confidence by design, as positional uncertainty in these cases is bounded by schematic and documentation fidelity rather than direct visual verification. Photo-guided cases (Domain B, *n* = 33) spanned a range from medium to high confidence depending on imaging quality, multi-angle availability, landmark identifiability, and photo-model concordance, with the majority of photo-guided cases achieving high marks on these items and supporting contact-level resolution. The highest-confidence subset comprised photo-guided cases in which a MiDaS depth map was successfully generated and applied (*n* = 7), as the depth map converts a two-dimensional photograph into an anchored surface representation that constrains electrode drape geometrically and reduces user variability in the cloth-simulation step. We note that MiDaS depth maps do not constitute a quantitative ground truth, and absolute scale is resolved by alignment to the patient-specific craniotomy model; rather, MiDaS provides an additional independent line of geometric evidence that, when combined with photo-model landmark concordance, supports the highest tier of localization confidence available within the pipeline (Supplementary data sheet 1).

### 3.4 Alignment in standard space

**Figure 7:**
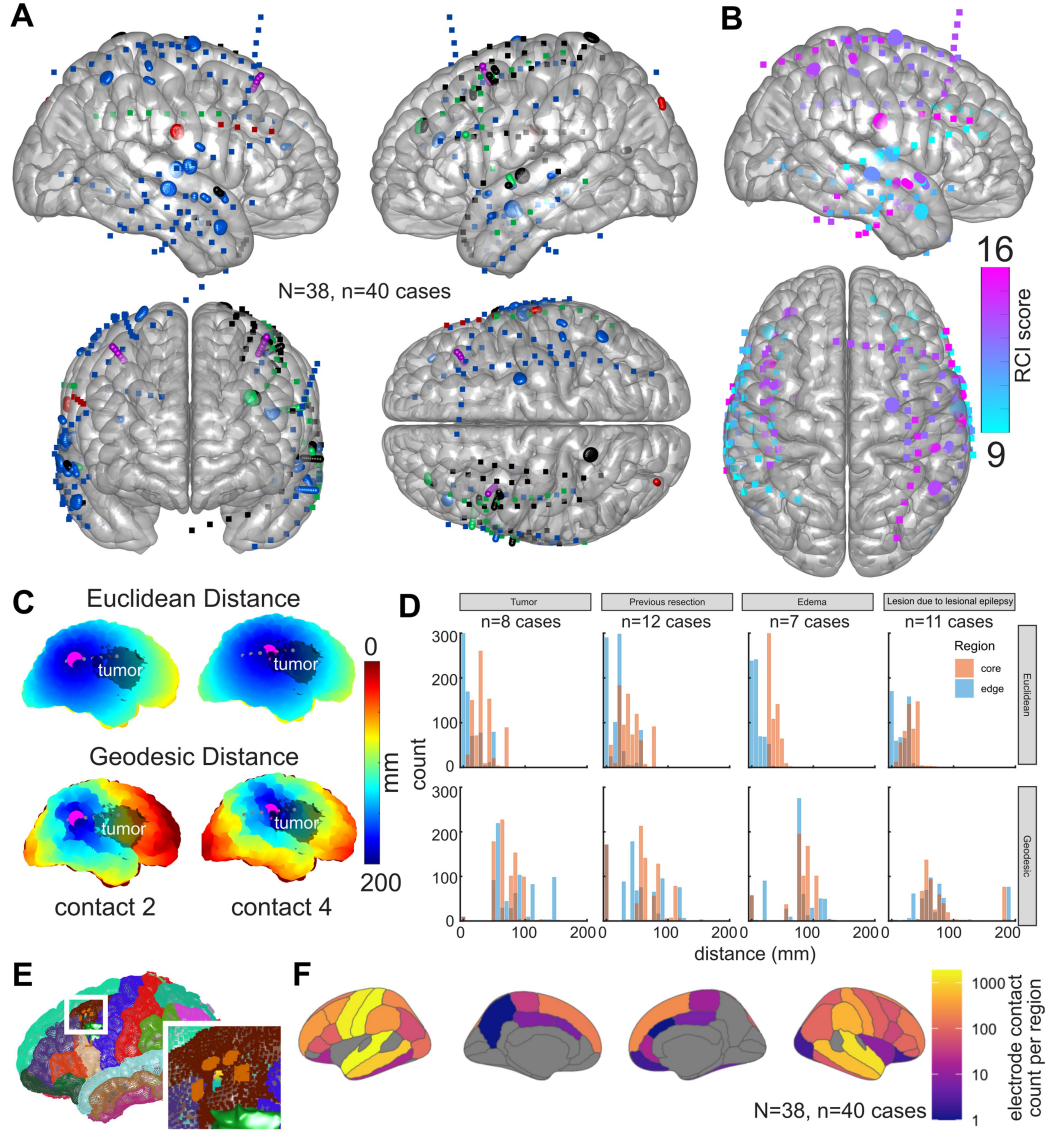
Electrode localizations and distances to pathology mapped to the MNI brain. **(A)** MNI localization for all cases, including epilepsy (blue), tumor (green, black, and red), and movement disorders (pink). **(B)** Each electrode location with the color coded based on reconstruction confidence index (RCI) score. **(C)** Visualizations of brain surface per-vertex Euclidean distance (top) and geodesic distance (bottom) for contact 2 (left) and contact 4 (right) of a clinical strip (selected contacts are magenta). Notably, the black region is the segmented tumor. **(D)** Per electrode contact distances to pathology across the data set, including only the data sets with photographic localization (domain B cases). **(E)** Example case of DKT mapping relative to a placed electrode (white box and inset image). **(F)** Localized counts of contacts across the data set and assignments to different identified DKT brain regions.

Transforming patient-specific reconstructions into MNI space using volumetric Fieldtrip tools (Oostenveld et al., 2011) allowed visualization of lesion and electrode distributions across the cohort (Figure 7A). This demonstrated consistent coverage of cortex despite heterogeneity in surgical sites, establishing the feasibility of group-level analyses. Further, to identify the regions covered by electrode localizations with higher confidence (high RCI scores) versus lower confidence (low RCI scores or Note-guided localizations), we could plot the RCI score on the common brain. This could inform how we consider the gathered data for future analyses (Figure 7B).

### 3.5 Distance measures and localization relative to brain regions

Euclidean and geodesic distances mapped neurophysiological signals to the locations, offering a spatial map of ongoing pathological dynamics. To illustrate the difference between geodesic and Euclidean distances on the brain, we included two electrode contact examples of these metrics on the brain in an individual case (Figure 7C). Not surprisingly, the distribution of distances to pathology increases with geodesic distances as the measure accounts for the curvature of the brain (Figures 7C,D). For instance, for the 8 cases where we could measure the distance to the tumor boundary, the mean and standard deviation of the Euclidean distance was 14.52 ± 15.638 mm, while the same for the Geodesic distance was 85.40 ± 32.064 mm. These values differed to the distances to the core (center of the tumor; Euclidean: 35.74 ± 17.803 mm; Geodesic: 74.24 ± 19.468 mm) (Figure 7D; Supplementary Table S4). In general, there was overlap between tumor and non-tumor cases in terms of distance to the pathology, indicating that this data set could be used to compare neural signals across neuropathologies while taking into account measured distances in comparing neural signals (Figure 7D; Supplementary Table S4). Note-guided cases (*n* = 7), for which electrode positions were inferred from clinical IOM documentation and atlas parcellation rather than photographic verification, were excluded from quantitative distance analyses, as their positional uncertainty is not bounded in the same manner as photo-guided cases. All distance measures reported in this section therefore reflect photo-guided cases only as there was at least photographic proof of the electrode location (*n* = 33).

### 3.6 Localization validation

**Figure 8:**
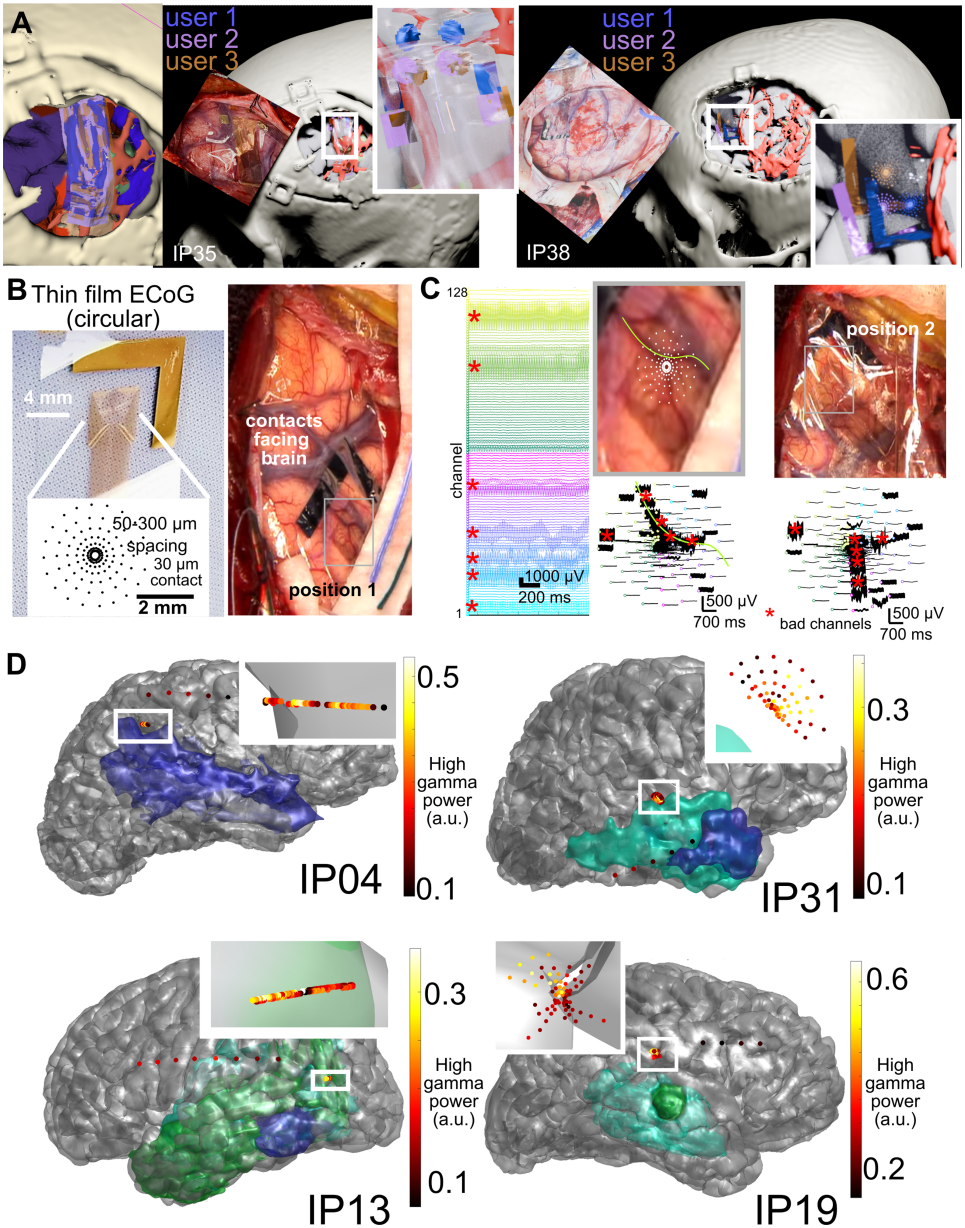
Localization validation and mapping of physiological signals to electrode positions. **(A)** Left: overlaid electrode positions in 3D slicer. Middle: 3D reconstruction of electrode positions by three different experienced users, color coding of user positions as blue, purple, and gold for case IP35. Right: electrode localizations by the same three users for IP38. Note: IP35 and IP38 are the same patient, but different surgical cases. **(B)** Left: close-up view of the circular thin film ECoG device which is placed on the brain, as shown in the photograph (right) in position 1. **(C)** Left: neurophysiological local field potential recordings, with each line color coded to indicate position as in the rightward plots. Note the bad channels are highlighted by red asterisks. **(C)** The recording with that position (position 1 as in **B** shows those same bad channels align with a vessel on the brain, lined by green in the top photograph and on the electrode map (bottom). Right: at a different position, different contacts are bad which likely correspond to underlying tissue relative to the electrode. **(D)** Examples of high gamma activity plotted as color on the electrode contacts in four different cases.

While there is not a current gold standard for identifying electrode locations intraoperatively, we performed a few steps of cross-validation. First, three experienced users localized electrodes in seven cases with high RCI scores to compare the locations of the electrodes across cases (Figure 8A). On a per-contact basis, the mean differences across the contacts were 4.062 ± 1.798 mm, ranging from 2.27 to 6.65 mm, median 3.2624 mm across 7 cases, including three positions (IP19) (Figure 8A).

These results, though with only a small data subset, could be important in taking into account this error involved in electrode localization. Therefore, further error correction may be helpful for fine localization. Therefore, in a small subset of cases, we found that the neural signal varied with the location, with high resolution thin film electrodes seemingly mapping to the underlying vasculature (Figures 8B,C). For a certain range of channels which were spatially localized, the recording signal became more noisy. This spatial distribution shifted with the electrode moving to another location, indicating the electrode contacts were not just bad channels for the duration of the recording (Figure 8C). Other approaches for validation could involve physiological maps of activity. While the coverage in these examples is low, we could also plot the high gamma power (70–200 Hz) at each contact, shown in four examples (Figure 8D). Future work could involve data sets with more extensive spatial coverage.

## 4 Discussion

This study introduces ALIGNER, a systematic open-source pipeline for reconstructing intraoperative surface electrode array placements and quantitatively mapping neural activity to patient-specific anatomy and pathology. A central contribution of this work is the creation of a framework that enables analysis of spatial gradients in neural activity relative to patient-specific pathology in intraoperative recordings, bridging a longstanding gap between transient surgical physiology and anatomically grounded structure–function analysis. By integrating multimodal imaging, cortical surface reconstructions, intraoperative photographs, and machine learning-based depth estimation, this approach overcomes the absence of reliable tools for anatomically precise localization of transient electrodes used during surgery. Whereas chronic implantation studies have benefited from post-implant CT and MRI coregistration or chronic and semichronic implants (Tadel et al., 2011; Dykstra et al., 2012; Groppe et al., 2017; Magnotti et al., 2020; Mercier et al., 2022; Felsenstein and Peled, 2017; Soper et al., 2022, 2023; Wang et al., 2023), intraoperative datasets have remained comparatively underexploited. Intraoperative cases present unique challenges: electrodes are placed only transiently, are rarely captured on imaging, and are often inconsistently documented (Rigolo et al., 2020; Bastos et al., 2021; Juvekar et al., 2023; Rasheed et al., 2025).

BrainTRACE (Oten et al., 2025) and features of RAVE (Magnotti et al., 2020) have represented some of many forays which could fill this gap, taking into account the photographs in an integrated GUI for electrode mapping. Ultimately, though, the method involved comparing photos or videos of the surgery which would not only include the brain surface but can, and likely should, include craniotomy and the electrode. Further, a difference to these other platforms is that, in our modular pipeline, we are placing a 3D model of the electrode onto the patient’s reconstructed brain within the craniotomy window using Blender and modeled physics (Community, 2018). Additionally, the model electrodes were built to scale, mimicking the movement of the electrodes as observed in the OR (Figures 4, 5).

ALIGNER directly addresses the mismatch between electrode technology development with available reproducible methods for localizing intraoperative placements by combining segmentation, physics-based modeling, and computer-vision depth estimation to reconstruct placements that would otherwise be lost to the surgical record. We did not need to build this software from scratch in a single platform. Instead, we took advantage of the strengths of each software separately (3D Slicer, Blender, FreeSurfer, etc.) and their interchangeable file formats to capture as much information as possible while using landmarks (craniotomy, vessels, depth maps, etc.) and 3D models to virtually reproduce the electrode placement in the operating room. This pipeline and its modularity then allowed us to incorporate novel devices as needed including high channel count electrodes (Paulk et al., 2024). Elements of this pipeline have already been applied to electrode placement in brain-computer interface contexts (Hadar et al., 2026), and its modularity positions it as a scalable foundation for future neurostimulation and implantable neurodevice applications requiring precise anatomical localization.

Another innovation of this framework is the adaptation of MiDaS depth estimation to neurosurgical imaging. Originally trained on LiDAR and stereo datasets for robotics and computer vision (Ranftl et al., 2021, 2022), MiDaS generates monocular depth cues that, when coupled with Blender-based physics simulations, produce anatomically plausible electrode seating on cortical surfaces. By employing this computer vision approach, the ALIGNER pipeline transforms intraoperative photographs from static documentation into quantitative data sources to anchor reconstructions with geometric spatial cues, with the possibility of moving into a dynamic space using video and object tracking tools and computer vision in the future (Mathis et al., 2018; Nath et al., 2019). This cross-disciplinary adaptation underscores the potential to leverage advances from other fields, such as computer vision and machine learning, for neuroscience applications, especially when conventional imaging modalities are limited.

A defining feature of the pipeline is its ability to facilitate distance-to-pathology relationships in human intraoperative recordings. Both Euclidean and geodesic distance measures can be extracted from reconstructions, allowing systematic electrophysiological analysis with respect to lesion anatomy. As a result, this pipeline could facilitate analysis that combines anatomically precise localization with spatially structured electrophysiological signatures that both recapitulate established observations and extend them to intraoperative human recordings, a domain where such gradients have traditionally remained obscured. Quantitative analyses that directly relate electrophysiological signal features to distance from pathology, drawing on the geodesic and Euclidean distance measures generated by this pipeline, form the basis of forthcoming work.

These analyses are especially pertinent for clinical practice due to their potential to refine intraoperative decision-making. Surgeons must frequently balance resection extent with preservation of eloquent cortex, relying on a combination of anatomical intuition, functional mapping, and electrophysiological recordings (Spena et al., 2010; You and Qiao, 2021). By linking recorded signals to quantitative measures of distance from pathology, ALIGNER provides an objective framework for delineating resection margins. In addition, similar analyses could inform prognosis by identifying the spatial extent of pathological network involvement, or guide adjunct therapies targeted to perilesional cortex. This pipeline may also serve as a framework for future extensions with intraoperative stimulation mapping or real-time visualization systems (Bastos et al., 2021; Haouchine et al., 2022; Barr et al., 2025; Frisken et al., 2025), allowing surgical teams to situate ongoing physiological data within the patient’s reconstructed anatomy. Indeed, a reason we decided to rely heavily on 3Dslicer is that there are currently powerful tools used for mapping tumors and close to real-time localization (Zhang et al., 2020; Juvekar et al., 2023; Safdar et al., 2023; Barr et al., 2025; Frisken et al., 2025). Further, the deformations that occur during resections may need to be modeled and mapped to the electrophysiology (Haouchine et al., 2020b; Haouchine et al., 2020a; Bastos et al., 2021; Yu et al., 2022; Juvekar et al., 2023; Frisken et al., 2025). Blender may allow for the types of brain and vascular shifts which occur during resective surgery through its physics engine originally built for 3D rendering animation (Community, 2018).

Beyond intraoperative electrophysiology, accurate anatomical reconstruction will become increasingly critical as neurostimulation technologies, implantable neurodevices, and brain–computer interfaces continue to advance, particularly as the field moves toward prescriptive, standardized, and personalized stimulation paradigms (Hadar et al., 2024, 2026). As stimulation-based therapies and closed-loop neural systems expand in both clinical and research settings, precise localization of electrodes relative to patient-specific anatomy will be essential for ensuring replicability, interpretability, and patient safety. Recent work in human neurostimulation has emphasized the need for anatomically grounded models to contextualize stimulation effects and to support reproducible mapping across individuals and institutions. In this context, the reconstruction framework presented here provides a scalable foundation for situating stimulation and recording interfaces within individualized anatomical and pathological landscapes, helping to bridge experimental neurotechnology with clinically responsible deployment.

At a research level, this methodology enables systematic interrogation of how focal lesions perturb neural networks across scales. Gliomas, for example, have been shown to form functional synapses with neurons, reshaping local circuit dynamics (Krishna et al., 2023; Taylor et al., 2023). Epileptic cortex, likewise, exhibits pathological high-frequency oscillations that propagate through vulnerable networks (Zijlmans et al., 2012). By anchoring intraoperative signals to precise anatomical coordinates, ALIGNER provides a robust process to link mechanisms to the oscillatory patterns observed during surgery. Moreover, transforming patient-specific reconstructions into MNI space facilitates normalized group-level analyses across diverse pathologies, enabling cross-subject comparison without sacrificing spatial specificity.

The robustness of the pipeline is further strengthened by the integration of SynthSR prior to cortical surface reconstruction in cases with distorted anatomy. Large lesions, edema, or mass effect can disrupt conventional FreeSurfer processing, leading to surface discontinuities or reconstruction failure. By incorporating SynthSR to generate anatomically consistent surrogate images, the pipeline expands applicability to non-ideal clinical imaging datasets and enhances generalizability across institutions. This addition is particularly relevant for real-world neurosurgical cohorts, where imaging quality and protocols vary widely. Importantly, the localization workflow is designed to be practical and efficient, relying entirely on open-source tools and modest computational resources, and can be completed on clinically realistic timescales even in cases with heterogeneous or incomplete data. The timing data presented in Section 3.2 further suggest that the most interface-dependent components of the pipeline can be learned and executed within a single supervised training session, supporting accessibility for new users.

The framework also highlights how reconstruction quality depends on intraoperative documentation. Reconstructions supported by photographic or video data, augmented with depth estimation, represent a higher-fidelity tier of localization than atlas- and note-guided inference alone. While the latter remains valuable when visual data are unavailable, this distinction underscores the importance of systematic intraoperative documentation for future research applications. Establishing standardized imaging or video capture protocols could substantially improve reconstruction accuracy and reproducibility across centers. Importantly, because ALIGNER is built entirely on open-source platforms (FreeSurfer, 3D Slicer, Blender, and MiDaS), it is widely accessible and adaptable. Researchers can easily integrate additional segmentation methods, alternative depth models, or real-time analysis modules, ensuring scalability as new tools emerge.

Importantly, the existence of low-confidence reconstructions does not diminish their scientific utility. Note-guided cases reliably localize electrodes to the correct lobe, supported by clinical IOM documentation, stimulation-based functional mapping, and coarse atlas parcellation. While these cases cannot support millimetric or contact-level analyses appropriate for high-confidence photo- and depth-map-guided reconstructions, they remain appropriate for research questions framed at the broad lobar or regional level, such as comparing neural activity between frontal and temporal cortex, characterizing oscillatory patterns within a specific anatomical territory, or pooling lobe-level signal across cohorts. The Reconstruction Confidence Index is intended to support this kind of pathway-appropriate analysis: rather than excluding cases that fall short of the highest fidelity tier, the framework allows researchers to match the spatial granularity of their analytic question to the spatial granularity supported by the reconstruction.

There are many avenues that future research can explore to build off this work. First, scaling the pipeline to larger, multi-institutional datasets will be essential to evaluate generalizability and to rigorously test spatial electrophysiological hypotheses. Integration with postoperative imaging and longitudinal outcome data could further link intraoperative physiology with clinical trajectories. Finally, embedding reconstruction and visualization tools into intraoperative workflows may ultimately bridge the gap between retrospective analysis and prospective surgical guidance. By transforming transient intraoperative recordings into rigorously reconstructed, anatomically grounded datasets, this pipeline advances both the methodological foundation and translational potential of human intraoperative electrophysiology.

### 4.1 Limitations

There are several key limitations to this pipeline and our data that should be considered when interpreting our findings. First, intraoperative video and images were available only for a subset of cases (*n* = 33 of 40 cases), which constrains the spatial precision of our electrode reconstructions in the remaining patients. While we utilized atlas and clinical note-guided inferences for placement in these cases, this process cannot fully substitute for direct visual confirmation and, thus, represents a lower-resolution localization approach than photo-guided reconstruction. Electrode localization in these cases cannot be verified to the same degree and support only regional rather than contact-level certainty. This distinction is reflected in the separate reporting of Domains B and C of the Reconstruction Confidence Index.

Moreover, the brain undergoes non-rigid intraoperative deformation, commonly termed brain shift, that is driven by loss of cerebrospinal fluid, gravity, and tissue manipulation (Haouchine et al., 2020a). The magnitude of this displacement can be substantial and is expected to be greater in cases involving larger craniotomies, significant CSF egress, or tumor debulking. ALIGNER does not currently incorporate explicit brain shift correction, and reconstructions therefore reflect the anatomy of the preoperative MRI rather than the intraoperative state which may be deformed. The use of intraoperative photographs captured at the time of recording partially mitigates this effect by anchoring electrode placement to the brain as it appears in the operating room but does not eliminate residual displacement between the reconstructed cortical surface and true intraoperative anatomy.

This dataset has sparse coverage of the brain with limited coverage of the occipital regions. More widespread coverage over multiple areas may be needed for future studies into the effects on recorded neural signals.

In cases where FreeSurfer recon-all failed due to prior resection, surgical cavities, or extensive scar tissue, preprocessing with SynthSR was used to generate anatomically plausible reconstructions. While the synthesized surface reconstruction of this approach enables parcellation and coordinate export in otherwise intractable cases, it introduces an additional source of uncertainty as the synthesized anatomy may deviate from the true underlying structure. This limitation is most relevant in regions with mass effect or perilesional distortion without complete tissue loss, where subtle discrepancies between reconstructed and actual anatomy may affect electrode localization. Electrode contacts in these regions should be interpreted with greater caution, particularly near large lesions. In contrast, regions distant from pathology are expected to be less affected by these transformations. As a result, cases requiring SynthSR are explicitly flagged within the Domain A score of the Reconstruction Confidence Index.

## 5 Conclusion

This study introduces a validated open-source pipeline for reconstructing intraoperative surface electrode arrays and integrating electrophysiological recordings with patient-specific anatomy. This framework leverages multimodal imaging, cortical surface modeling, depth estimation, and physics-based simulation to achieve anatomically grounded localization of transient intraoperative electrodes. As a result, it addresses a critical methodological gap that has limited the systematic use of intraoperative recordings in clinical and research domains.

Our pilot cohort of patients with epilepsy and brain tumors demonstrates the feasibility and biological plausibility of this approach. The demonstrated ability to interrogate distance-dependent electrophysiological signals underscores the potential of this pipeline to reveal spatially resolved structure–function relationships that would remain hidden in traditional electrode reconstructions.

This work carries notable clinical and scientific implications. Clinically, the pipeline establishes a foundation for future integration of electrophysiological signals with three-dimensional models of patient-specific pathology and vasculature; this information could guide intraoperative decision-making with spatially grounded context. In addition, this pipeline enables systematic investigation into how focal lesions reshape cortical networks, bridging cellular-level mechanisms such as hyperexcitability and neuron–glioma coupling with macroscale oscillatory dynamics. Because the framework is built entirely on open-source platforms, it can be readily adopted, scaled, and refined across institutions.

Future iterations of this pipeline should focus on expanding the analytic scope and testing generalizability. Integration with intraoperative stimulation mapping and high-frequency oscillation analyses could provide convergent markers of pathological cortex, while coupling reconstructions to postoperative outcomes would enable longitudinal validation of intraoperative signatures. Scaling to larger, multi-institutional cohorts will be essential to confirm the reproducibility of spatially structured gradients observed in this pilot. By transforming transient intraoperative recordings into rigorously reconstructed and anatomically grounded datasets, this framework offers a path to link cellular-level mechanisms of hyperexcitability and tumor–neuron coupling with macroscale surgical physiology, advancing both neurosurgical practice and systems neuroscience.

## Data Availability

The data that support the findings of this study are available from the corresponding author upon reasonable request and agreement to only receive de-identified data. Documentation and code including commands for different packages are available at the main repository (https://github.com/Center-For-Neurotechnology/BrainInterface3D). Electrode tracking over the cortical surface involved 3D reconstruction of the brain surface and segmentation including vessels using 3Dslicer (https://www.slicer.org/) (Kikinis and Pieper, 2011; Fedorov et al., 2012), Freesurfer (https://surfer.nmr.mgh.harvard.edu/) (Dale et al., 1999; Fischl and Dale, 2000; Fischl et al., 2001; Desikan et al., 2006; Dykstra et al., 2012; Felsenstein and Peled, 2017; Billot et al., 2021, 2022; Iglesias et al., 2021; Soper et al., 2022, 2023), and the Human Connectome Project (HCP) pipeline (https://github.com/Washington-University/HCPpipelines) (Glasser et al., 2013, 2016; Elam et al., 2021). The segmentations and surface reconstructions along with the skull was imported into Blender (https://www.blender.org/) (Community, 2018) and electrode locations were exported using custom python code. Geodesic distance measures were made possible with pymeshlab (https://github.com/cnr-isti-vclab/PyMeshLab).

## Footnotes

1
https://www.fieldtriptoolbox.org/

2
http://surfer.nmr.mgh.harvard.edu

3
https://www.humanconnectome.org/

4
https://mangoviewer.com/mango.html

5
https://github.com/rordenlab/dcm2niix

6
https://surfer.nmr.mgh.harvard.edu/fswiki/SynthSR

7
https://surfer.nmr.mgh.harvard.edu/fswiki/SynthSeg

8
https://github.com/PerkLab/SlicerFreeSurfer

9
https://github.com/netstim/SlicerANTs

10
https://github.com/Center-For-Neurotechnology/BrainInterface3D

11
https://github.com/isl-org/MiDaS

12
https://www.fieldtriptoolbox.org/

13
https://github.com/cnr-isti-vclab/PyMeshLab
