# Supplementary material for: A modular and flexible pipeline for intraoperative electrode reconstruction and localization in patients with brain lesions
Source: Front Neural Circuits. 2026 Jun 10;20:1814667. doi: 10.3389/fncir.2026.1814667 (PMC13290761; doi:10.3389/fncir.2026.1814667)
Supplement: Supplementary file 2 [file Supplementary_file_1.docx]

**Supplemental Material for:**

**A Modular and Flexible Pipeline for Intraoperative Electrode Reconstruction and Localization in Patients with Brain Lesions**

**Haziq Rabbani^1,2,3^ & Ipsita Das,^2,3^ Ayan S. Mandal,^2,3^ Thomas Nelson,^4^ Peter Hadar,^2,3^ Brian Hsueh,^2,5^ Roberto Ciordia,^2^ Brian F. Coughlin,^2,3^ Emory Peng,^2,3^ Daniel R. Cleary,^6^ Kelly L. Collins,^6^ Ahmed M. T. Raslan,^6^ Ziv M. Williams,^5^ Bryan D. Choi,^5^ G. Rees Cosgrove,^5^ Shadi A. Dayeh,^7^ Pamela S. Jones,^5^ Harmanpreet K. Tiwana,^2^ Gavin Dunn,^5^ R. Mark Richardson,^5^ Wenya Linda Bi,^5^ Steven Tobochnik,^8^ Sydney S. Cash,^2,3^ Daniel P. Cahill,^5^ Angelique C. Paulk^2,3^**

^1^ College of Medicine, Northeast Ohio Medical University, Rootstown, OH 44272, USA

^2^ Department of Neurology, Mass General Brigham, Boston MA 02114, USA

^3^ Center for Neurotechnology and Neurorecovery, Boston MA 02114, USA

^4^ Division of Neuro-Oncology, Department of Neurosurgery, University of California San Francisco, San Francisco CA 94143, USA

^5^ Department of Neurosurgery, Mass General Brigham, Boston MA 02114, USA

^6^ Department of Neurosurgery, Oregon Health State University, Portland OR 97239, USA

^7^ Department of Neurosurgery, University of California San Diego, La Jolla, CA 92093, USA

^8^ Center for Neuro-Oncology, Department of Medical Oncology, Dana-Farber Cancer Institute, Boston MA 02215, USA

*** Correspondence:**Angelique C. Paulk
apaulk@mgh.harvard.edu

Keywords: human, intraoperative, epilepsy, tumor, imaging, electrophysiology, parcellation.

**Table S1. Diagnosis, age, and lobe and hemisphere coverage.** Participant designations include previously published designations with only 4 cases not previously published (Paulk et al., 2021, 2022; Yang et al., 2021; Coughlin et al., 2023; Tan et al., 2023; Lee et al., 2024). Repeated designation pairs indicate separate surgical cases from the same individual: IP13 and IP34 correspond to one participant, and IP35 and IP38 correspond to another participant.

| **Designation** | **Surgical Indication** | **Age at Surgery** | **Sex** | **Brief Diagnosis** | **Brain Region** | **Affected/ Recorded Hemisphere** |
| --- | --- | --- | --- | --- | --- | --- |
| IP01 | Epilepsy | 33 | M | Temporal lobe epilepsy from previous tumor diagnosis | Temporal | Left |
| IP05 | Epilepsy | 37 | F | Temporal lobe epilepsy | Temporal | Right |
| IP08 | Tumor | 22 | M | Glioma,IDH wt | Temporal | Right |
| IP09 | Epilepsy | 24 | M | Temporal lobe epilepsy | Temporal | Right |
| IP10 | Epilepsy | 43 | M | Epilepsy due to stroke | Frontal | Right |
| IP14 | Epilepsy | 29 | F | Focal cortical Dysplasia Type IIIb | Temporal | Right |
| IP16 | Epilepsy | 44 | F | Temporal lobe epilepsy | Temporal | Right |
| IP17 | Epilepsy | 39 | F | Temporal lobe epilepsy | Temporal | Right |
| IP02 | Tumor | 37 | F | Glioma,IDH mutant | Temporal | Left |
| IP03 | Epilepsy | 40 | F | Temporal lobe epilepsy | Temporal | Right |
| IP04 | Epilepsy | 42 | F | Temporal lobe epilepsy | Temporal | Right |
| IP06 | Epilepsy | 25 | F | Temporal lobe epilepsy from meningioma | Temporal | Right |
| IP07 | Tumor | 62 | M | Glioma,IDH wt | Temporal | Left |
| IP11 | Tumor | 39 | M | Glioma,IDH mutant | Temporo-frontal-parietal | Right |
| IP12 | Epilepsy | 36 | F | Temporal lobe epilepsy | Temporal | Left |
| IP13,IP34 | Epilepsy | 22 | F | Temporal lobe epilepsy | Temporal | Right |
| IP15 | Malformation | 52 | F | Cavernous malformation | Temporal | Left |
| IP18 | Epilepsy | 29 | M | Focal cortical Dysplasia | Frontal | Right |
| IP19 | Tumor | 55 | M | metastatic carcinoma | Temporal | Right |
| IP23 | Epilepsy | 48 | F | Temporal lobe epilepsy from meningioma | Temporal | Right |
| IP25 | Epilepsy | 33 | M | Hemorrhagic leukoencephalitis | Frontal | Right |
| IP27 | Tumor | 52 | M | Glioma,IDH wt | Frontal | Left |
| IP28 | Tumor | 32 | F | Glioma,IDH mutant | Frontal | Left |
| IP31 | Epilepsy | 52 | M | Post Traumatic Epilepsy | Temporal | Right |
| IP32 | Epilepsy | 57 | M | Temporal lobe epilepsy | Temporal | Right |
| IP33 | Epilepsy | 22 | M | Congenital insult and mesial temporal sclerosis | Temporal | Left |
| IP13,IP34 | Epilepsy | 24 | F | Temporal lobe epilepsy | Temporal | Right |
| IP35, IP38 | Tumor | 64 | F | Glioma,IDH wt | Frontal | Left |
| IP35, IP38 | Tumor | 64 | F | Glioma,IDH wt | Frontal | Left |
| IP36 | Epilepsy | 55 | F | Temporal lobe epilepsy | Temporal | Left |
| IP37 | Epilepsy | 36 | F | Focal cortical Dysplasia | Frontal | Left |
| Pt03 | Epilepsy | 54 | F | Temporal lobe epilepsy | Temporal | Left |
| Pt01 | Movement Disorder | 63 | F | Parkinson's Disease | Frontal | Right |
| Pt02 | Movement Disorder | 75 | M | Parkinson's Disease | Temporo-parietal | Left |
| HP01 | Epilepsy | 26 | M | Neurofibromatosis Type 1 | Temporal | Left |
| HP02 | Epilepsy | 48 | F | Temporal lobe epilepsy | Temporal | Left |
| Prt01 | Epilepsy | 58 | M | Temporal lobe epilepsy | Temporal | Right |
| Prt02 | Tumor | 28 | F | Glioma,IDH mutant | Temporal | Left |
| Prt03 | Tumor | 50 | M | Glioblastoma | Parietal | Left |
| Prt04 | Tumor | 68 | F | Glioblastoma | Temporal | Left |

**Table S2.**

**Distribution of recording devices and concurrent clinical electrodes across cases.** Recording devices included PEDOT:PSS microelectrodes and Neuropixels probes with multiple device designs. X channel strip electrodes denote X-contact strip electrodes with 1 cm spacing.

| **Designation** | **Surgical Indication** | **Electrode Type** | **Electrode Design** | **Clinical Electrode** | **Photo or video** | **RCI Score** |
| --- | --- | --- | --- | --- | --- | --- |
| IP01 | Epilepsy | PEDOT:PSS | 50 micron pitch 2 column | 6 channel strip, 6 contact depth | No | 12 |
| IP05 | Epilepsy | PEDOT:PSS | 800 micron pitch 2 column | 6 channel strip, contact depths | No | 11 |
| IP08 | Tumor | PEDOT:PSS | 50 micron pitch 2 column | 4 channel strip, 6 channel depth | No | 14 |
| IP09 | Epilepsy | PEDOT:PSS | 50 micron pitch 2 column | 6 channel strip, 6 channel strip | Yes | 13 |
| IP10 | Epilepsy | PEDOT:PSS | 50 micron pitch 2 column | 6 channel strip, 6 channel strip | Yes | 9 |
| IP14 | Epilepsy | PEDOT:PSS | 50 micron pitch 2 column | 4 channel strip, 4 channel depth | Yes | 12 |
| IP16 | Epilepsy | PEDOT:PSS | 50 micron pitch 2 column | 4 channel strip, 4 channel depth | Yes | 15 |
| IP17 | Epilepsy | PEDOT:PSS | Circular grid | 4 channel strip, 6 channel depth | Yes | 10 |
| IP02 | Tumor | PEDOT:PSS | 50 micron pitch 2 column | 6 channel strip | No | 16 |
| IP03 | Epilepsy | PEDOT:PSS | 50 micron pitch 2 column | 8 channel strip, 8 channel depth, 8 channel depth | Yes | 9 |
| IP04 | Epilepsy | PEDOT:PSS | 50 micron pitch 2 column | 4 channel strip, 8 channel depth, 4 channel depth | Yes | 16 |
| IP06 | Epilepsy | PEDOT:PSS | 50 micron pitch 2 column | 8 channel strip, 20 channel grid | Yes | 15 |
| IP07 | Tumor | PEDOT:PSS | 50 micron pitch 2 column | 8 channel strip | Yes | 12 |
| IP11 | Tumor | PEDOT:PSS | 50 micron pitch 2 column | 8 channel strip | Yes | 13 |
| IP12 | Epilepsy | PEDOT:PSS | 50 micron pitch 2 column | 6 channel strip | Yes | 14 |
| IP13,IP34 | Epilepsy | PEDOT:PSS | 50 micron pitch 2 column | none | Yes | 13 |
| IP15 | Malformation | PEDOT:PSS | 50 micron pitch 2 column | 8 channel strip | Yes | 11 |
| IP18 | Epilepsy | PEDOT:PSS | Circular grid | 8 channel strip | Yes | 13 |
| IP19 | Tumor | PEDOT:PSS | Circular grid | 4 channel strip | Yes | 16 |
| IP23 | Epilepsy | PEDOT:PSS | Circular grid | none | Yes | 12 |
| IP25 | Epilepsy | PEDOT:PSS | Circular grid | 8 channel strip | Yes | 14 |
| IP27 | Tumor | PEDOT:PSS | 50 micron pitch 2 column | 8 channel strip | Yes | 14 |
| IP28 | Tumor | PEDOT:PSS | Circular grid | 8 channel strip | Yes | 14 |
| IP31 | Epilepsy | PEDOT:PSS | Circular grid | 8 channel strip | Yes | 11 |
| IP32 | Epilepsy | PEDOT:PSS | Circular grid | 8 channel strip | Yes | 11 |
| IP33 | Epilepsy | PEDOT:PSS | Circular grid | 64 channel grid | Yes | 11 |
| IP13,IP34 | Epilepsy | PEDOT:PSS | Circular grid | 16 channel grid (4 x 4) | Yes | 11 |
| IP35, IP38 | Tumor | PEDOT:PSS | 50 micron pitch 2 column | none | Yes | 14 |
| IP35, IP38 | Tumor | PEDOT:PSS | 50 micron pitch 2 column | 8 channel strip, depth | Yes | 16 |
| IP36 | Epilepsy | PEDOT:PSS | 50 micron pitch 2 column | 8 channel strip | Yes | 11 |
| IP37 | Epilepsy | PEDOT:PSS | Circular grid | 8 channel strip, 4 channel strip | Yes | 10 |
| Pt03 | Epilepsy | Neuropixels | Neuropixels, checkerboard | none | Yes | 12 |
| Pt01 | Movement Disorder | Neuropixels | Neuropixels, checkerboard | none | No | 11 |
| Pt02 | Movement Disorder | Neuropixels | Neuropixels, checkerboard | none | No | 11 |
| HP01 | Epilepsy | PEDOT:PSS | PEDOT short depth | 6 channel strip | Yes | 12 |
| HP02 | Epilepsy | PEDOT:PSS | PEDOT short depth | 8 channel strip, 4 channel strip | Yes | 14 |
| Prt01 | Epilepsy | Neuropixels | Neuropixels, checkerboard | 8 channel strip, 4 channel depth, 8 channel depth | No | 8 |
| Prt02 | Tumor | Neuropixels | Neuropixels, checkerboard | 8 channel strip | No | 10 |
| Prt03 | Tumor | Neuropixels | Neuropixels, checkerboard | 8 channel strip | No | 10 |
| Prt04 | Tumor | Neuropixels | Neuropixels, checkerboard | 8 channel strip | No | 9 |

**Table S3. Imaging details.** Participant designations include previously published designations with only 4 cases not previously published (Paulk et al., 2021, 2022; Yang et al., 2021; Coughlin et al., 2023; Tan et al., 2023; Lee et al., 2024). Repeated designation pairs indicate separate surgical cases from the same individual: IP13 and IP34 correspond to one participant, and IP35 and IP38 correspond to another participant. MRI information included here is for the preoperative structural T1 and for the postoperative CT where available (or a postoperative MRI was used for craniotomy reconstruction).

| **Designation** | **MRI pixel spacing, x (mm)** | **MRI pixel spacing, y (mm)** | **MRI slice thickness (mm)** | **MRI magnetic strength** | **CT pixel spacing, x (mm)** | **CT pixel spacing, y (mm)** | **CT slice thickness (mm)** |
| --- | --- | --- | --- | --- | --- | --- | --- |
| IP01 | 0.9766 | 0.9766 | 1 | 3 | - | - | - |
| IP05 | 0.976563 | 0.976563 | 1 | 3 | - | - | - |
| IP08 | 0.4297 | 0.4297 | 5 | 1.5 | - | - | - |
| IP09 | 0.976563 | 0.976563 | 1 | 3 | 0.435547 | 0.435547 | 0.5 |
| IP10 |  |  |  |  | 1 | 1 | 1 |
| IP14 | 0.954545 | 0.954545 | 1 | 3 | - | - | - |
| IP16 | 0.4297 | 0.4297 | 1.4 | 1.5 | - | - | - |
| IP17 | 0.7031 | 0.7031 | 2 | 3 | - | - | - |
| IP02 | 1 | 1 | 1 | 3 | 0.400391 | 0.400391 | 0.5 |
| IP03 | 1 | 1 | 1 | 3 | 1 | 1 | 1 |
| IP04 | 0.938 | 0.938 | 1 | 3 | 1 | 1 | 1 |
| IP06 | 1 | 1 | 1 | 1.5 | 1 | 1 | 1 |
| IP07 | 0.859 | 0.859 | 1.8 | 3 | 0.402 | 0.402 | 2.5 |
| IP11 | 0.9375 | 0.9375 | 0.9 | 3 | 0.7 | 0.7 | 0.7 |
| IP12 | 1 | 1 | 1 | 3 | 1 | 1 | 1 |
| IP13,IP34 | 0.352 | 0.352 | 1.015 | 3 | 1 | 1 | 1 |
| IP15 | 1 | 1 | 1 | 3 | - | - | - |
| IP18 | 1 | 1 | 1 | 3 | - | - | - |
| IP19 | 1 | 1 | 1 | 3 | 0.7 | 0.7 | 0.7 |
| IP23 | 1 | 1 | 1 | 3 | 0.449218 | 0.449218 | 4.97431 |
| IP25 | 0.938 | 0.938 | 0.9 | 3 | 1 | 1 | 1 |
| IP27 | 1 | 1 | 1 | 3 | - | - | - |
| IP28 | 0.7 | 0.7 | 0.7 | 3 | - | - | - |
| IP31 | 1 | 1 | 1 | 3 | 1 | 1 | 1 |
| IP32 | 1 | 1 | 1 | 3 | 1 | 1 | 1 |
| IP33 | 1 | 1 | 1 | 3 | 1 | 1 | 1 |
| IP13,IP34 | 1 | 1 | 1 | 3 | 1 | 1 | 1 |
| IP35, IP38 | 0.4883 | 0.4883 | 0.500001 | 3 | 0.449219 | 0.449219 | 0.6 |
| IP35, IP38 | 0.4883 | 0.4883 | 0.500001 | 3 | 0.449219 | 0.449219 | 2 |
| IP36 | 1 | 1 | 1 | 3 | 1 | 1 | 1 |
| IP37 | 1 | 1 | 1 | 3 | 1 | 1 | 1 |
| Pt03 | 0.7 | 0.7 | 0.7 | 3 | 0.443 | 0.443 | 0.6 |
| Pt01 | 0.7 | 0.7 | 0.7 | 3 | 0.463 | 0.463 | 0.6 |
| Pt02 | 0.7 | 0.7 | 0.7 | 3 | 0.463 | 0.463 | 0.6 |
| HP01 | 0.43 | 0.43 | 1 | 3 | 0.443 | 0.443 | 0.6 |
| HP02 | 0.9 | 1 | 0.9 | 3 | 0.443 | 0.443 | 0.6 |
| Prt01 | 0.9375 | 0.9375 | 4.955124 | 3 | 0.472656 | 0.472656 | 0.599976 |
| Prt02 | 1 | 1 | 1 | 3 | 0.472656 | 0.472656 | 0.599976 |
| Prt03 | 1 | 1 | 1 | 3 | 0.488281 | 0.488281 | 0.625 |
| Prt04 | 1 | 1 | 1 | 3 | 0.429688 | 0.429688 | 0.6 |

**Table S4. Distance Measures from the electrode contacts and pathologies**. Distance metrics for the different cases relative to the varied pathologies.

| **Distance measure** | **Number of cases included** | **Mean distance (mm)** | **Distance standard deviation (mm)** |
| --- | --- | --- | --- |
| Distance to the tumor edge, Euclidean distance | 8 | 14.524 | 15.638 |
| Distance to the tumor core, Euclidean distance | 8 | 35.737 | 17.803 |
| Distance to the previous resection edge, Euclidean distance | 12 | 19.796 | 17.187 |
| Distance to the previous resection core, Euclidean distance | 12 | 41.656 | 18.831 |
| Distance to the edema edge, Euclidean distance | 7 | 11.523 | 8.411 |
| Distance to the edema core, Euclidean distance | 7 | 38.233 | 8.040 |
| Distance to the non-tumor lesion edge, Euclidean distance | 11 | 18.678 | 12.435 |
| Distance to the non-tumor lesion core, Euclidean distance | 11 | 34.592 | 11.240 |
| Distance to the tumor edge, Geodesic distance | 8 | 85.405 | 32.064 |
| Distance to the tumor core, Geodesic distance | 8 | 74.236 | 19.468 |
| Distance to the previous resection edge, Geodesic distance | 12 | 54.000 | 38.116 |
| Distance to the previous resection core, Geodesic distance | 12 | 60.532 | 35.499 |
| Distance to the edema edge, Geodesic distance | 7 | 73.415 | 35.929 |
| Distance to the edema core, Geodesic distance | 7 | 81.021 | 27.671 |
| Distance to the non-tumor lesion edge, Geodesic distance | 11 | 83.927 | 47.953 |
| Distance to the non-tumor lesion core, Geodesic distance | 11 | 85.813 | 44.447 |


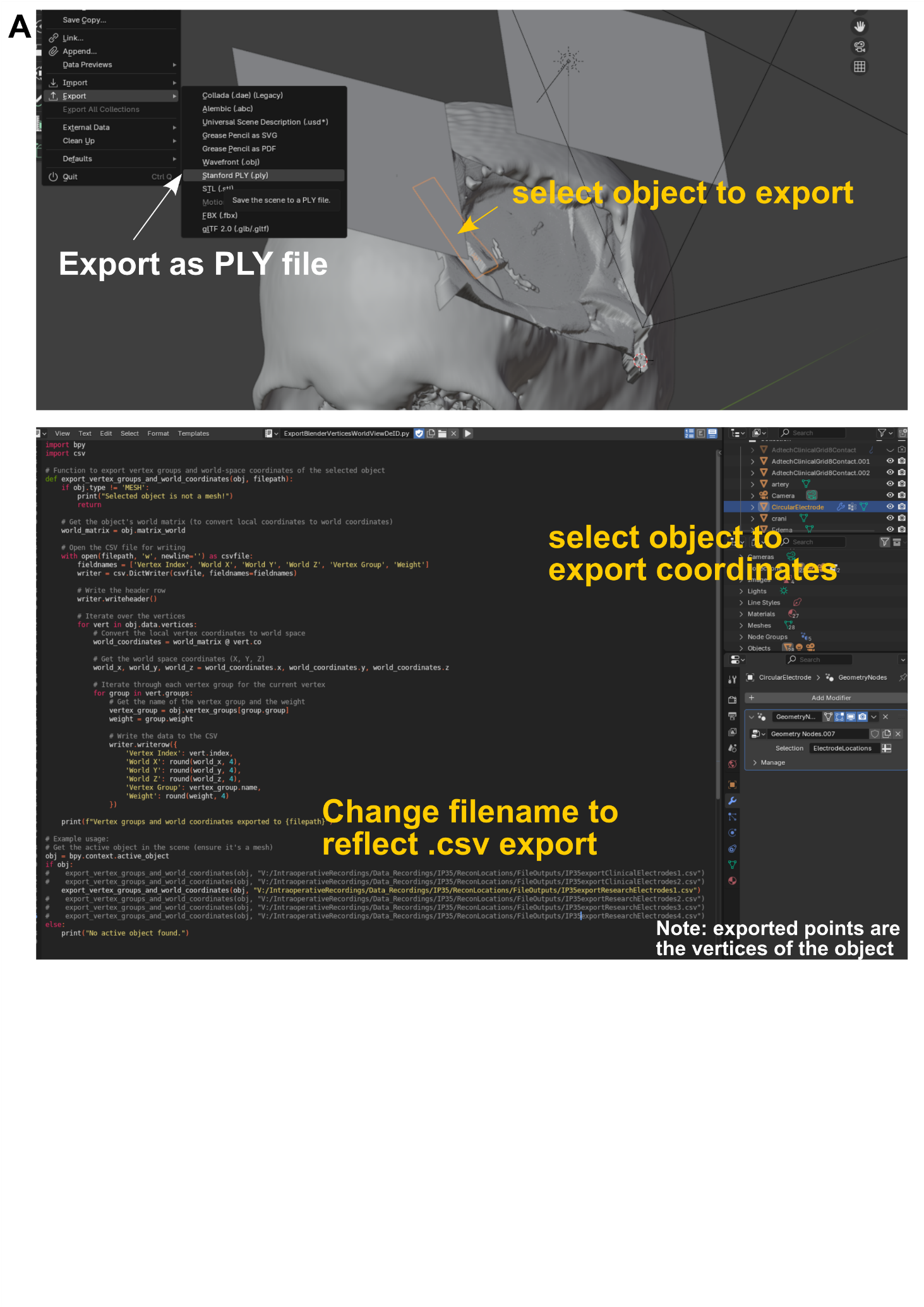


**Supplemental Figure 1. Examples of exporting placed electrodes into PLY (A) or .csv (B) files for later data processing**.

**References**

Coughlin, B., Muñoz, W., Kfir, Y., Young, M. J., Meszéna, D., Jamali, M., et al. (2023). Modified Neuropixels probes for recording human neurophysiology in the operating room. *Nature Protocols*. doi: 10.1038/s41596-023-00871-2

Lee, K., Paulk, A. C., Ro, Y. G., Cleary, D. R., Tonsfeldt, K. J., Kfir, Y., et al. (2024). Flexible, scalable, high channel count stereo-electrode for recording in the human brain. *Nature Communications* 15, 218. doi: 10.1038/s41467-023-43727-9

Paulk, A. C., Kfir, Y., Khanna, A. R., Mustroph, M. L., Trautmann, E. M., Soper, D. J., et al. (2022). Large-scale neural recordings with single neuron resolution using Neuropixels probes in human cortex. *Nature Neuroscience* 25, 252–263. doi: 10.1038/s41593-021-00997-0

Paulk, A. C., Yang, J. C., Cleary, D. R., Soper, D. J., Halgren, M., O’Donnell, A. R., et al. (2021). Microscale Physiological Events on the Human Cortical Surface. *Cerebral Cortex* 31, 3678–3700. doi: 10.1093/cercor/bhab040

Tan, H., Paulk, A. C., Stedelin, B., Cleary, D. R., Nerison, C., Tchoe, Y., et al. (2023). Intraoperative application and early experience with novel high-resolution, high-channel-count thin-film electrodes for human microelectrocorticography. *Journal of Neurosurgery*, 1–12. doi: 10.3171/2023.7.JNS23885

Yang, J. C., Paulk, A. C., Salami, P., Heon Lee, S., Ganji, M., Soper, D. J., et al. (2021). Microscale dynamics of electrophysiological markers of epilepsy. *Clinical Neurophysiology* 32, 2916–2931. doi: https://doi.org/10.1016/j.clinph.2021.06.024
